# Transcriptome analysis of a respiratory *Saccharomyces cerevisiae *strain suggests the expression of its phenotype is glucose insensitive and predominantly controlled by Hap4, Cat8 and Mig1

**DOI:** 10.1186/1471-2164-9-365

**Published:** 2008-07-31

**Authors:** Nicklas Bonander, Cecilia Ferndahl, Petter Mostad, Martin DB Wilks, Celia Chang, Louise Showe, Lena Gustafsson, Christer Larsson, Roslyn M Bill

**Affiliations:** 1 School of Life and Health Sciences, Aston University, Aston Triangle, Birmingham, B4 7ET, UK; 2 Department of Chemical and Biological Engineering/Molecular Biotechnology, Chalmers University of Technology, Box 462, 405 30, Göteborg, Sweden; 3 Mathematical Sciences, Chalmers University of Technology and Göteborg University, SE-412 96, Göteborg, Sweden; 4 Cameron International Ltd., Queen Street, Stourton, Leeds, LS10 1SB, UK; 5 The Wistar Institute, 3601 Spruce Street, Philadelphia, Pennsylvania, 19104, USA

## Abstract

**Background:**

We previously described the first respiratory *Saccharomyces cerevisiae *strain, KOY.TM6*P, by integrating the gene encoding a chimeric hexose transporter, Tm6*, into the genome of an *hxt *null yeast. Subsequently we transferred this respiratory phenotype in the presence of up to 50 g/L glucose to a yeast strain, V5 *hxt1-7*Δ, in which only *HXT1-7 *had been deleted. In this study, we compared the transcriptome of the resultant strain, V5.TM6*P, with that of its wild-type parent, V5, at different glucose concentrations.

**Results:**

cDNA array analyses revealed that alterations in gene expression that occur when transitioning from a respiro-fermentative (V5) to a respiratory (V5.TM6*P) strain, are very similar to those in cells undergoing a diauxic shift. We also undertook an analysis of transcription factor binding sites in our dataset by examining previously-published biological data for Hap4 (in complex with Hap2, 3, 5), Cat8 and Mig1, and used this in combination with verified binding consensus sequences to identify genes likely to be regulated by one or more of these. Of the induced genes in our dataset, 77% had binding sites for the Hap complex, with 72% having at least two. In addition, 13% were found to have a binding site for Cat8 and 21% had a binding site for Mig1. Unexpectedly, both the up- and down-regulation of many of the genes in our dataset had a clear glucose dependence in the parent V5 strain that was not present in V5.TM6*P. This indicates that the relief of glucose repression is already operable at much higher glucose concentrations than is widely accepted and suggests that glucose sensing might occur inside the cell.

**Conclusion:**

Our dataset gives a remarkably complete view of the involvement of genes in the TCA cycle, glyoxylate cycle and respiratory chain in the expression of the phenotype of V5.TM6*P. Furthermore, 88% of the transcriptional response of the induced genes in our dataset can be related to the potential activities of just three proteins: Hap4, Cat8 and Mig1. Overall, our data support genetic remodelling in V5.TM6*P consistent with a respiratory metabolism which is insensitive to external glucose concentrations.

## Background

We engineered the first respiratory *Saccharomyces cerevisiae *strain, KOY.TM6*P, by integrating the gene encoding a chimeric hexose transporter, Tm6*, into the genome of an *hxt *null yeast [1]. Subsequently we demonstrated the transferability of this respiratory phenotype to a yeast strain, V5 *hxt1-7*Δ, in which only *HXT1-7 *had been deleted [2]. The resulting V5.TM6*P strain produced only minor amounts of ethanol when cultured on external glucose concentrations as high as 5% [2]. Despite the fact that the glucose flux was reduced to 30% in the V5.TM6*P strain compared with that of its parental V5 strain, the V5.TM6*P strain produced biomass at a specific rate as high as 85% of that produced by the V5 wild-type strain, the yield itself increasing by 50% in the mutant compared to its parental strain.

Having performed this initial physiological characterization of the V5.TM6*P strain, we wanted to examine more thoroughly the basis of its respiratory phenotype by comparing its transcriptome with that of its wild-type parent strain, V5. This approach has been widely used to obtain a global picture of the cellular responses of yeast to a wide range of physiological changes, including those experienced at the diauxic shift [3-5]. Recently Ohlmeier and colleagues [6] demonstrated that major changes at the transcriptional level are not reflected at the protein level. Indeed, focusing on the mitochondrial proteome following a diauxic shift, they showed that the levels of only 18 out of 253 identified proteins had changed (17 increased, and 1 decreased). Among them were proteins involved in the tricarboxylic acid (TCA) cycle (Sdh1, Sdh2, and Sdh4) and the respiratory chain (Cox4, Cyb2, and Qcr7). This seeming disagreement with their observations of heterogeneous changes in the transcriptome (where more than 4,000 changes were recorded) is also consistent with our own prior data where we showed that the protein patterns obtained from a 2D-PAGE analysis of the two strains (grown under the same conditions used in this study) were not substantially different [2]. We did note, however, that the levels of the upper-part glycolytic intermediates and ATP did differ between the two strains, and that Cdc19 is present in lower amounts in V5.TM6*P. This discrepancy between proteome and transcriptome regulation has also been observed by others [7] and is also consistent with the fact that there is an acknowledged lack of relationship between the levels of glycolytic proteins and the glycolytic flux [8-10]. Nonetheless, researchers continue to depend on mRNA as an indicator of cellular state, which is a situation that will continue while methods for the global analysis of protein expression are improved [7].

In this respect, Brauer and colleagues [3-5] have used transcriptome analysis to examine adjustments and remodelling of the metabolism of glucose-limited yeast cultures. In that study, the authors constructed metabolic models consistent with their cDNA array data. In this work, the V5.TM6*P strain was therefore characterized at the transcriptome level in comparison with the parental V5 strain in order to obtain a more global understanding of any genetic remodelling underlying the physiological differences between them. Both strains were cultured in 50 g/L glucose with six samples being taken at defined points between 38 and 6 g/L glucose for cDNA array analysis in two independent fermentations for each strain. Consequently, we generated a highly redundant dataset (n = 12) where 922 genes were either induced or repressed when comparing V5 and V5.TM6*P under all glucose concentrations. Using *in vivo *studies together with documented binding specificities of three transcription factors known to have a role in the regulation of respiratory genes (the Hap complex, Cat8 and Mig1), it was possible to correlate the up-regulation of all but 18 of the 146 genes induced by a factor ≥2.0 with the presence of one or more of their binding sites. This means that 75% of the transcriptional response of the genes in our dataset could be related to the activities of just three transcription factors, which in turn would account for 88% of the response of the induced set. Finally, we made the unexpected observation that both the induction and repression of many of the genes in our dataset had a clear glucose dependence in the parent V5 strain that was not present in V5.TM6*P. This important result indicates that the relief of glucose repression in the wild-type is already operable at much higher glucose concentrations than is currently widely accepted and that the phenotype of the respiratory strain is glucose insensitive by comparison.

## Results

We compared the transcriptional profiles of the V5 and V5.TM6*P strains cultured in 50 g/L glucose, with samples extracted between 38 and 6 g/L residual glucose. We identified 190 genes with adjusted p-values < 0.05 that changed their expression levels either up by a factor of ≥2.0 (146 genes) or down by a factor of ≤0.5 (44 genes) when comparing the two strains at all three sampling points. These genes are presented by functional category of their gene products in Additional file 1 and are discussed below. Additional file 2 provides the information in Excel format and also includes other relevant literature data. A further 636 genes changed their expression levels either up by a factor of <2.0 (317 genes) or down by a factor > 0.5 (319 genes) and are presented as part of Additional file 3. The remaining 96 genes were either dubious, Ty-transposable elements, or were detected due to cross-hybridisation, as indicated in the legend to Additional file 1.

### The majority of genes that have an altered expression in V5.TM6*P compared with its parental strain also have an altered expression following diauxic shift

We compared our dataset with three sets of expression data for yeast undergoing a diauxic shift, where the samples had been taken at least 2 hours after the glucose is depleted [3-5]. Of the 190 genes in our dataset that changed their expression levels either up or down by a factor of 2 or more, 154 of these could be identified as changing in the same direction in at least one of these three datasets, with 124 having at least two matches. Only 8 genes (*ERG3*, *RPS27A*, *YHB1*, *TRX1*, *YER188w*, *YKL066w*, *YAR068w *and *Y0L150c*) had changes that were in the opposite direction in at least two of the datasets. This significant overlap between our data set and the data sets of the three previous studies is entirely consistent with the fact that there is a transition from a respiro-fermentative metabolism in V5 to a respiratory metabolism in V5.TM6*P.

### Glycolytic and gluconeogenic genes have substantially altered expression levels in V5.TM6*P

**Figure 1:**
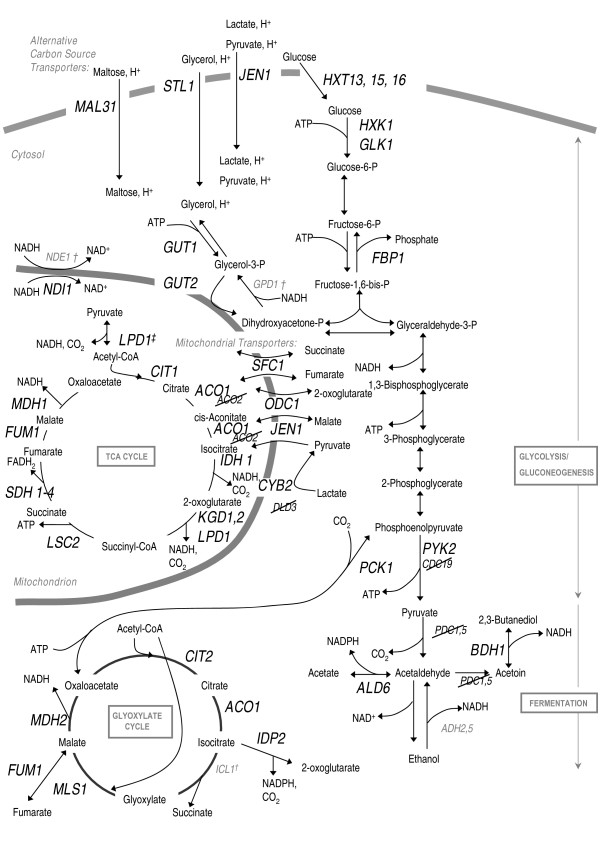
**An overview of our dataset in the context of key yeast metabolic pathways**. Genes with altered expression in V5.TM6*P compared with V5 are marked as follows: induced genes are denoted in large, bold font; down-regulated genes are crossed out. Genes that were previously found to be altered in studies of respiratory-state yeast [6], but which are not found in our dataset are denoted in small, grey text. The chimeric Tm6*p transporter, which comprises the amino-terminal half of Hxt1 and the carboxy-terminal half of Hxt7, is responsible for the respiratory phenotype of V5.TM6*P at high glucose concentrations and is present in the plasma membrane. The grey boundaries represent the plasma membrane (top) and the mitochondrial membrane (left). ^†^Note that *ICL*(1.5), *NDE1 *(1.8) and *GPD1 *(1.8) are induced in our study (see Additional file 3). ^‡ ^*LPD1 *is a component of pyruvate dehydrogenase.

Four of the most strongly induced genes in Additional file 1 are the glycolytic genes, *HXK1 *(factor 9.0) and *GLK1 *(3.8), and the gluconeogenic genes, *PCK1 *(6.9) and *FBP1 *(5.3). Indeed, as shown in Figure 1, the induction in V5.TM6*P of several glycolytic and gluconeogenic genes could be consistent with these cells accommodating for a lack of glucose; the hypothesized mechanism behind the strain's reduced ethanol-producing, respiratory phenotype. The final step in glycolysis which produces pyruvate, is catalyzed by pyruvate kinase. In V5.TM6*P the pyruvate kinase genes, *CDC19 *and *PYK2*, respond in an opposing manner: *CDC19 *is repressed by a factor of 0.4 (consistent with our earlier proteome analysis where Cdc19 is present in lower amounts in V5.TM6*P than V5) while *PYK2 *is induced and is relieved of its glucose-repressed state as a result of the low glucose flux in the respiratory strain. Reduced ethanol production in V5.TM6*P compared to the parental strain is also consistent with the two most strongly repressed genes *PDC1 *(0.2) and *PDC5 *(0.2) encoding the main pyruvate decarboxylases.

### Alternative carbon source usage genes are induced in V5.TM6*P compared with V5, consistent with a reduced glucose flux

An adaptation to enable growth on alternative carbon sources is seen in the induction of the plasma membrane transporter genes *MAL31 *and *STL1 *as well as the genes *SUC2*, *GUT1*, *GUT2*, *ALD6 *and *BDH1 *encoding proteins for the utilization of sucrose, glycerol, acetaldehyde and butanediol, respectively. *GUT1 *in particular is strongly induced by a factor of 6.2, and *STL1 *is the most strongly induced of all genes on the cDNA array (21.7), suggesting that V5.TM6* has undergone a substantial genetic adjustment compared to its parent. Furthermore, *CYB2 *(10.1) and *DLD3 *(0.5), whose gene products are involved in the conversion of lactate to pyruvate, and in the case of *CYB2 *feeding the released electrons into the electron transport chain, are oppositely regulated. Biochemical data and sequence patterns for *YGL157w *(0.3) [11], *YAL061w *(2.9) and *DSF1 *(9.4, homologous to D-mannitol 2-dehydrogenase from *Rhodobacter sphaeroides) *suggest that these ORFs encode an oxidoreductase, a polyol dehydrogenase and a putative sugar dehydrogenase, respectively, which could be involved in channelling alternative carbon sources into the TCA cycle. The carbon-deficient state of V5.TM6*P [1] could also be consistent with the induction of *CRC1 *and *CAT2 *whose gene products are involved in *β*-oxidation and carnitine-dependent transport of acetyl-CoA from peroxisomes to mitochondria as a way of using carbon from sources other than glucose [12]. The induction of *SFC1*, *JEN1*, *FBP1 *and *ATO2 *seen in our study has previously been reported to be a specific identifier of carbon-limited growth [13].

### All TCA- and glyoxylate cycle genes have increased expression in V5.TM6*P compared with V5

The genes encoding the enzymes of the TCA cycle (*CIT1*, *ACO1, IDH1*, *KGD1*, *KGD2*, *LSC2*, *SDH1-4*, *FUM1*, *MDH1*) all have a significant (adjusted p-value < 0.05) increase in expression when comparing V5 and V5.TM6*P. In addition, the gene encoding an isoform of aconitase (*ACO2*) has its expression level reduced by a factor of 0.5. Fermentation is almost completely abolished in the V5.TM6*P strain, and we have previously shown that the TCA cycle has a higher carbon flux [2] to harness the electron flow into the respiratory chain and generate a proton gradient using F_0_F_1_-ATPase for ATP synthesis (several respiratory chain genes are induced, as discussed below). Interestingly, all genes of the TCA-cycle are induced by greater than a factor of 3.5 except for *CIT1*/*CIT2 *and *KGD1*/*KGD2 *where the factor change is 2.2 – 3.5 for each gene of the pair, presumably yielding the same overall transcriptional response. Consistent with this, Ohlmeier and colleagues have shown that Sdh1, 2 and 4 all have increased expression after a diauxic shift [6].

The glyoxylate cycle is anabolic, yielding gluconeogenic precursors, and all its genes (*CIT2*, *ACO1, ICL1 *(induced by a factor of 1.5; Additional file 3), *MLS1 *and *MDH2*) are induced in V5.TM6*P compared with V5 (Additional file 1, Fig. 1). This could be consistent with the correspondingly increased biomass yield of the V5.TM6*P strain (Table 1). We note that V5.TM6*P has a lower biomass yield than expected from a totally respiratory phenotype. In chemostat culture under aerobic, fully respiratory conditions, a yield of 0.5 g/g is typical at low dilution rates, whereas the observed low yield in V5.TM6*P has already been published using a range of sugars and concentrations [2]. Furthermore, numerous experiments on KOY.TM6*P, our original respiratory strain based on CEN.PK2-1C, [1] have yielded identical results. These observations are in agreement with our findings in Table 1 of 0.32 g/g in V5.TM6*P and 0.21 g/g in V5 from batch cultures.

**Table 1:** Growth characteristics of V5 and V5.TM6*P at 30°C starting at 50 g/L glucose.

	**V5**	V5.TM6*P
**Generation time during glucose growth (h)**	3.7 (0.2; n = 2)	4.5 (0.3; n = 4)
**Biomass yield (g/g)**	0.21 (0.02 ; n = 2)	0.32 (0.01; n = 4)
**Glucose consumption (mmolg dry weight)^-1^h^-1^**	9.00 (0.70; n = 2)	2.70 (0.20; n = 4)
**Ethanol yield (g/g)**	0.33 (0.01; n = 2)	0.05 (0.01; n = 4)
**Glycerol yield (g/g)**	0.04 (0; n = 2)	0

Yields were calculated as g product (dry weight, ethanol or glucose, respectively)/g of glucose consumed/catabolised for V5 and V5.TM6*P, cultured as described in 'Materials and Methods'. The numbers in parentheses are the standard error of the mean, together with the number of replicates (n) for each experiment. The seemingly low biomass yield for V5 was measured after consumption of all glucose. Yield increases as all ethanol and glycerol are consumed during subsequent respiratory growth.

### Genes encoding respiratory chain enzymes are induced in V5.TM6*P compared with V5

**Figure 2:**
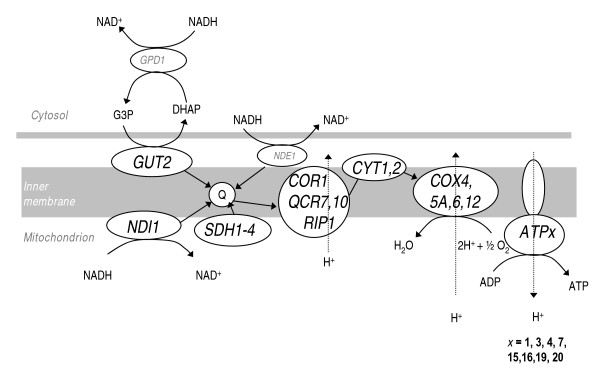
**An overview of our dataset in the context of the respiratory chain in yeast**. Genes with altered expression in V5.TM6*P compared with V5 are marked as follows: genes induced by a factor ≥2.0 are denoted in large, bold font; genes induced by a factor <2.0 are denoted in smaller, grey font.

33 key genes encoding mitochondrial enzymes involved in respiration – as well as F_0_F_1_-ATPase and its chaperones – are all induced by a factor of 2 – 4 in V5.TM6*P (Additional file 1). The gene encoding Ndi1 (Fig. 2), which transfers electrons from NADH to ubiquinone (Q in Fig. 2) is induced the most (by a factor of 4.1), while all other components of the respiratory chain are induced by a factor of 2 – 2.5. Genes encoding heme synthesis and incorporation (*HEM2*, *CYT2*), complex assembly (*MBA1*) and heme degradation (*HMX1*) are also induced by a factor of 2 – 2.5. Cytochrome c oxidase also has different chaperones that are induced to form an active structure by proteolysis (*COX20*), as well as delivery of copper (*COX17*). In spite of the higher respiratory rate in V5.TM6*P, several genes involved in detoxification of oxygen radicals are repressed, *e.g. *genes encoding peroxiredoxin (*AHP1*) and thioredoxin (*TRX2*) are repressed by a factor of 0.4 – 0.5. Conversely, the capacity for nitrous oxide detoxification is increased by the induction of *YHB1 *by a factor of 2.4. It is possible that the respiratory phenotype of V5.TM6*P could induce a higher level of oxygen radical formation inside its mitochondria and thus oxidation of guanine could occur on the mitochondrial DNA. Such detrimental effects could be counteracted, *via *the observed induction of *OGG1 *by a factor of 2.2.

### Changes of expression in genes encoding mitochondrial and plasma membrane transporters are consistent with a respiratory phenotype in V5.TM6*P

The genes encoding the hexose transporters, Hxt1 to Hxt7, have very high sequence identities [14] and thus cross reactivity between them is expected on the array. This explains the observed signal for *HXT1, 4 *and *6*, which have been deleted in V5.TM6*P. *HXT13*, *HXT15 *and *HXT16 *are known to be repressed by high levels of glucose and are consequently induced as expected in V5.TM6*P. A carbon source deficiency triggers an up-regulation of genes encoding plasma membrane proteins responsible for the import of alternative carbon sources, as already noted: *STL1 *(21.7; glycerol); *JEN1 *(10.2; lactate), *PUT4 *(5.8; proline – the main nitrogen source in grape juice) and *MAL31 *(3.1; maltose). A gene associated with nutrient deficiency in yeast [15], *YGP1*, is induced by a factor of 2.6, again supporting the notion that V5.TM6*P has undergone a genetic adjustment compared to its parent.

It seems that V5.TM6*P also has an altered requirement for metal ions in that genes encoding three metal ion channels have altered expression: *CTR3 *(copper transport) is induced by a factor of 3.0, while *ZRT1 *(zinc transport) and *ENB1 *(iron transport) are repressed by a factor of 0.4 – 0.5. It is likely that copper import is increased so that copper can be transferred to cytochrome c oxidase, which is the main copper-requiring enzyme in Additional file 1, with 3 copper ions per complex and a copy number of 5,000–12,000 per cell. There are only 45 genes in Additional file 1 with greater than 5,000 copies per cell and none of these has copper as a prosthetic group except cytochrome c oxidase.

The amino acid uptake systems encoded by *BAP3 *and *GNP1 *are repressed while the proline permease encoded by *PUT4 *is induced. *PUT4 *has a carbon source responsive element (CSRE) [16] that is expected to be induced at the low glucose flux experienced in the V5.TM6*P strain, while *BAP3 *and *GNP1 *are regulated by a sensor of extracellular amino acid concentration, where Ssy5 [17] is a vital component. It is likely that in V5.TM6*P the repression of *BAP3 *and *GNP1 *is a result of the repression (by a factor 0.65; Additional file 3) of *SSY5 *seen on the cDNA array. Interestingly, induction of genes encoding the ammonia transport system in the V5.TM6*P strain (*ATO2*, *MEP1*) could suggest that the strain is experiencing general nutrient limitation. It has previously been reported for example that the induction of another *MEP *family member, *MEP2*, is associated with nitrogen limitation [13].

### 88% of genes induced in V5.TM6*P compared to its parent have transcription factor binding sites for one or more of the Hap complex, Cat8 or Mig1

Genome-wide studies have yielded a wealth of information on which promoters are bound by different transcription factors [18]. In order to rationalise these resources, we began by looking for biologically-verified data on transcription factors that might control the repression of the subset of 44 genes of Additional file 1. Since only limited information was publicly available, we did not pursue this part of the dataset further. In contrast, for the 146 induced genes in Additional file 1, our observation of the similarity between the transcriptional changes in the V5 to V5.TM6*P transition and those in the transition from glucose to ethanol growth lead us to analyse the roles of Hap4, Cat8 and Mig1 which have been previously been verified to be crucial to the expression of genes in the diauxic shift [16,19,20]: Hap4 (in complex with Hap2, 3 and 5) [19] and Cat8 (optionally in complex with Sip4 and Adr1) [21] are transcriptional activators, whereas Mig1 (optionally with Mig2/3) is a transcriptional repressor that works in concert with Tup1-Cyc8 [22].

The Hap complex is comprised of proteins Hap2, 3, 4 and 5, and has separate subunits for DNA binding and transcriptional activation. DNA binding is mediated by Hap2, 3 and 5 [23] while Hap4 functions as an activation domain. The expression of *HAP4 *is repressed by glucose and is responsible for regulation of Hap complex target genes. We began by scrutinising data for a *HAP4 *over-expression strain, in which 246 genes had their expression affected by at least a factor of 2 [19], which is 5.3% of the genome. 56 of these genes were found in our dataset and are listed in Table 2. Only three of these (*GLK1*, *CTR3 *and *ZRT1*) are down-regulated in the *HAP4 *over-expression strain, whereas *GLK1 *and *CTR3 *are induced in the V5.TM6*P strain. Interestingly, a typical Hap complex binding site was not seen in the 500 nucleotide region upstream of the *GLK1, CTR3 *and *ZRT1 *start codon (where the first A in ATG is position 1) while all other genes in Table 2 except *AGX1*, *MNP1 *and *YNK1 *were found to have at least one Hap complex binding site. These Hap complex-binding-site-containing genes comprise a significant subset (26% of our dataset of 190 genes in Additional file 1) with a verified biological dependence on Hap4 activation. Clearly, then, Hap4 has a statistically-significant role to play in the metabolism of V5.TM6*P as there is an association between Hap4 dependence and our dataset (χ^2 ^= 182.75, degrees of freedom = 1, p = 0.00).

**Table 2:** Biologically-verified data on transcription factors that act on genes also found in Additional file 1: transcript dependence on *HAP4 *over-expression.

**ORF**	**GENE**	FACTOR CHANGE IN GENE EXPRESSION FROM V5 TO V5.TM6*P	**FACTOR CHANGE IN GENE EXPRESSION FROM WT TO A *HAP4 *OVER-EXPRESSION STRAIN **[19]
**Glycolysis**			

*YCL040W*	*GLK1*	3.8	0.4

**Alternative carbon source utilization**			

*YML054C*	*CYB2*	10.1	5.0

**TCA cycle**			

*YIL125W*	*KGD1*	2.2	2.2
*YDR148C*	*KGD2*	3.5	4.4
*YFL018C*	*LPD1*	2.0	2.2
*YKL085W*	*MDH1*	3.6	2.2
*YKL148C*	*SDH1*	4.6	4.7
*YLL041C*	*SDH2*	4.1	3.9
*YKL141W*	*SDH3*	3.7	2.3
*YDR178W*	*SDH4*	5.3	3.2

**Glyoxylate cycle**			

*YIR029W*	*DAL2*	3.0	3.0

**Respiratory chain**			

*YPL078C*	*ATP4*	2.1	2.0
*YKL016C*	*ATP7*	2.3	2.4
*YPR020W*	*ATP20*	2.5	3.4
*YGR174C*	*CBP4*	2.8	2.1
*YBL045C*	*COR1*	2.0	2.0
*YGL187C*	*COX4*	2.0	2.3
*YNL052W*	*COX5A*	2.0	2.1
*YHR051W*	*COX6*	2.1	2.2
*YLL009C*	*COX17*	2.8	4.7
*YOR065W*	*CYT1*	2.5	2.3
*YKL087C*	*CYT2*	2.2	2.8
*YIL098C*	*FMC1*	2.5	2.3
*YDL181W*	*INH1*	2.2	5.5
*YBR185C*	*MBA1*	2.3	2.3
*YML120C*	*NDI1*	4.1	4.4
*YEL024W*	*RIP1*	2.3	2.4
*YPR151C*	*SUE1*	2.2	3.0

**Plasma membrane transport**			

*YLL052C*	*AQY2*	3.0	3.0
*YLR411W*	*CTR3*	3.0	0.5
*YGL255W*	*ZRT1*	0.5	0.1

**Mitochondrial transport**			

*YNR002C*	*ATO2*	3.8	3.8
*YKL217W*	*JEN1*	10.3	2.9
*YPL134C*	*ODC1*	6.3	3.3

**Transcriptional regulation**			

*YOL071W*	*EMI5*	2.1	2.9
*YNL333W*	*SNZ2*	3.0	6.4

**Fatty acid metabolism**			

*YKL150W*	*MCR1*	3.1	2.8

**Biosynthesis**			

*YFL030W*	*AGX1*	3.6	7.8

**Ribosomal proteins in the mitochondria or cytosol**			

*YDR296W*	*MHR1*	2.0	2.5
*YGL068W*	*MNP1*	2.1	2.4
*YDR116C*	*MRPL1*	2.6	3.5
*YKL138C*	*MRPL31*	2.2	2.8
*YGR165W*	*MRPS35*	2.0	2.7
*YHR038W*	*RRF1 (FIL1)*	2.2	4.8
*YDR041W*	*RSM10*	2.1	2.4
*YDR175C*	*RSM24*	2.4	2.9
*YFR049W*	*YMR31*	2.5	2.6

**Other**			

*YBR262C*	*AIM5*	2.3	2.1
*YFR011C*	*AIM13*	2.1	3.4
*YLR168C*	*AIM30*	2.6	3.3
*YML087C*	*AIM33*	4.2	11.6
*YBR230C*	*OM14*	3.3	2.4
*YDL104C*	*QRI7*	2.1	2.7
*YOR187W*	*TUF1*	2.3	2.4
*YKL067W*	*YNK1*	3.1	2.1

**Not characterized**			

*YGR110W*	N/A	2.3	2.0

Genes were tabulated and sorted according to their roles in yeast cellular physiology, as described in the legend for Additional file 1. Note that the seven hexose transporter genes, *HXT1*-*7*, are deleted in the V5.TM6*P with the amino-terminal half of *HXT1 *and the carboxy-terminal half of *HXT7 *reincorporated as the TM6* chimera. In the 'Factor Change' column, change is expressed as a factor, where that factor is x when a gene expressed with intensity '1' in V5 is expressed with intensity 'x' in V5.TM6*P. All T-tests were jointly adjusted for multiple testing using the false discovery rate method (disregarding correlations between genes): p-values were adjusted so that when selecting all genes with p-values less than a threshold q, a proportion of q false positives would be expected amongst these genes. The genes shown have p-values < 0.05, and thus the expected false discovery rate is 5%. The factor in the final column is taken from Lascaris *et al *[19]. ^†^Note that due to overall high homology within the hexose transporter family, changes were still observed on the cDNA array for *HXT4 *(*YHR092C*) even though it is deleted as already mentioned.

**Figure 3:**
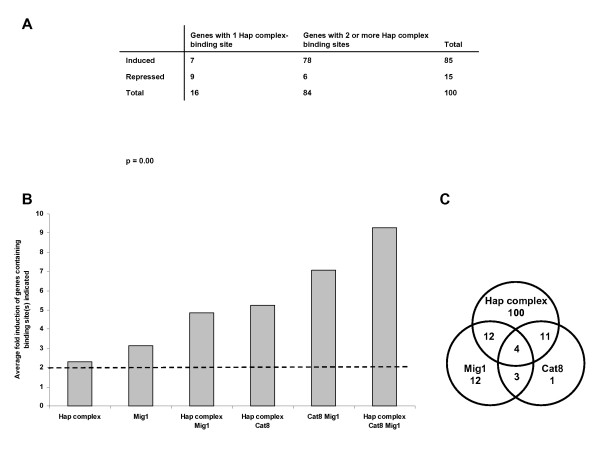
**(A) The influence of multiple Hap complex sites on the change in gene expression of the genes in Additional file **1. A Fisher's exact test was performed for genes containing predicted binding sites for the Hap complex and their association with induction or repression. Genes were grouped into either those with one Hap complex binding site or those with two or more according to Additional file 1. **(B) The influence of Hap4, Cat8 and Mig1 on the magnitude of induction of the genes in Additional file **1. The average fold-induction of genes in Additional file 1, grouped according to the transcription factor binding sites they contain, was calculated. It was observed that those containing binding sites for each of the Hap complex (where Hap4 is the activator), Cat8 and Mig1, had the highest average fold induction with Mig1 being a dominant factor in high induction. A bar for Cat8 is not included as only one gene (*YOR019W*, factor change 2.1) has a Cat8 site alone. **(C) A Venn diagram of the distribution of binding sites for the Hap complex, Cat8 and Mig1 in the genes in Additional file **1. 88% of the genes in Additional file 1 contain a binding site for one or more of the Hap complex, Cat8 and Mig1. The range of induction for the 190 genes from Additional file 1 is 2.0 to 21.7.

Since six of the genes from the *HAP4 *over-expression study did not contain Hap complex binding sites, we did a further *in silico *analysis of the induced genes of Additional file 1. We noted that the genes of Table 2, contained motifs that could be sub-divided into 2 distinct families: **CCAAT**G (which we denoted as a Hap4_1 site) and (G/**C)CAA**(G/**T**)CAA (a Hap4_2 site). The bold sequences are the conserved sequences previously identified in the *HAP4 *over-expression strain study [19]. The genes of Additional file 1 were then examined 500 nucleotides upstream and 200 nucleotides downstream from the start codon using WebMOTIF [24] for these two biologically-relevant sites since it has been previously demonstrated that Hap complex binding sites are statistically over-represented up to 400 nucleotides upstream from the start codon [19]: we found that 112/146 (77%) induced genes and 127/190 (67%) of the complete dataset in Additional file 1 had one or more Hap complex binding site (Additional files 1 and 4). Our analysis included presumed binding sites such as CAAATC and CCAAAC which arose from the biologically-verified data on transcription factors (Table 2). For example, *AQY2 *is identified as Hap complex-dependent, and has two CCAAAC sites, but no CCAATNA sites. There are only two other genes that do not contain CCAATNA sites in Table 2, namely *CRC1 *and *ALD6*. In addition it was notable that Hap complex consensus sequences are predominantly found upstream of mitochondrial genes with 84/127 (66%) of such genes having at least a partial mitochondrial location. Furthermore, of the induced genes with a known cellular location and at least two Hap complex binding sites, 69/80 (86%) are localized to the mitochondria. It appears, then, that Hap4 activation is preferentially directed at mitochondrially-related functions especially within the TCA-cycle and respiratory chain, but cytosolic support via the glyoxylate cycle is also potentially regulated by Hap4 activation (Additional file 1). It was further noted that multiple Hap complex binding sites are associated with induction (Fig. 3A). A Fisher's exact test showed that there was a statistically-significant association between the change in gene expression and the number of Hap complex binding sites in the gene (p = 0.00). This supported the fact that genes with two or more Hap complex binding sites are likely to be induced, whereas those with one site may be either induced or repressed.

In order to adopt a similar approach for Cat8, we examined available biological data for Cat8 and Adr1 (as they are co-regulators [25]) from chromatin immunoprecipitations [25], and mRNA expression ratios in wt/Δcat8 strains [16]. We identified 20 genes out of 66 from these studies that were also in our dataset (Table 3). Only *ATO2 *was oppositely regulated when comparing our dataset. For all other genes, the change in expression followed the same trend of up-regulation (Table 3). As for the Hap4 analysis, we examined these 20 genes for Cat8 binding sites, and identified a typical Cat8 motif [21] in 13 of them, nine being of the Cat8-Sip4 type, TCCATTSRTCCGR (Additional file 4). The remaining six induced genes (*CYB2*, *GUT1*, *GUT2*, *LSC2*, *SUE1 *and *ODC1*) were further scrutinised, and it was apparent that all except *ODC1 *had been identified from immunoprecipitation data for Adr1 alone, consistent with their lack of Cat8-Sip4 motif. The induced genes of Additional file 1 were therefore searched from the start codon to 1,500 nucleotides upstream for the generic Cat8 motif, CC------CCG motif giving 54 genes (Additional file 4). The non-coding intergenic regions of these genes were then searched using WebMOTIF [24] and *HXK1*, *COX4*, *ODC1*, *OM14*, *OM45 *and *YOR019w *were found to share the consensus sequence GCCSSTSS(W/Y))CMS in common with *IDP2 *and are thus also listed in Additional file 4. We noted that genes with these Cat8 binding sites are induced in V5.TM6*P by an average factor of 6.2 while the average for induced genes in Additional file 1 is 3.3. The highly-induced, Cat8-site-containing genes include *HXK1 *(factor change 9.0), *PCK1 *(6.9), *STL1 *(21.7), *JEN1 *(10.3), *ODC1 *(6.3) and *SFC1 *(18.3).

**Table 3:** Biologically-verified data on transcription factors that act on genes also found in Additional file 1: transcript dependence on Cat8 and Adr1.

**ORF**	**GENE**	FACTOR CHANGE IN GENE EXPRESSION FROM V5 TO V5.TM6*P^a^	**FACTOR CHANGE IN GENE EXPRESSION USING DATA FROM CHROMATIN IMMUNOPRECIPITATION FOR Cat8^b^**[25]**, mRNA EXPRESSION RATIOS FOR WT/Δcat8^c^**[16]** AND IMMUNOPRECIPITATION DATA FOR Adr1^d ^**[25].
**Gluconeogenesis**			

*YLR377C*	*FBP1*	5.3	5.0^b^; MAX^c^
*YKR097W*	*PCK1*	6.9	7.7^b^; 151^c^

**Alternative carbon source utilization**			

*YPL061W*	*ALD6*	2.3	5.2^b^; 8^c^
*YML054C*	*CYB2*	10.1	7.4^d^
*YHL032C*	*GUT1*	6.2	3.7^d^
*YIL155C*	*GUT2*	3.3	4.6^d^

**TCA cycle**			

*YGR244C*	*LSC2*	2.9	5.2^d^

**Glyoxylate cycle**			

*YCR005C*	*CIT2*	2.2	1.7^c^
*YLR174W*	*IDP2*	4	2.9^b^; 23^c^
*YOL126C*	*MDH2*	3	12.7^b^; 3.6^c^
*YNL117W*	*MLS1*	3.5	8.9^b^; 79^c^

**Respiratory chain**			

*YPR151C*	*SUE1*	3.7	4.4^d^

**Plasma membrane transport**			

*YOR348C*	*PUT4*	5.8	2.7^b^; 6.1^c^; 2.0^d^
*YDR536W*	*STL1*	21.7	4.4^c^

**Mitochondrial transport**			

*YNR002C*	*ATO2*	3.8	0.33^c^
*YML042W*	*CAT2*	2.6	2.8^b^; 2.4^c^
*YOR100C*	*CRC1*	2.2	8.1^c^
*YKL217W*	*JEN1*	10.3	3.6^b^; 10^c^; 10.5^d^
*YPL134C*	*ODC1*	6.3	3.2^b^
*YJR095W*	*SFC1*	18.3	6.2^b^; MAX^c^

The table is arranged as for Table 2. Factor changes denoted '^a^' are from Additional file 1, '^b^' refer to chromatin immunoprecipitation data for Cat8 [25], '^c^' refer to mRNA expression ratios for wt/*Δcat8 *[16] and '^d^' refer to chromatin immunoprecipitation data for Adr1 [25].

We observed that some strongly-induced genes in Additional file 1 did not have Hap complex or Cat8 binding sites. Indeed, the expression of many genes such as *SUC2 *is already known to be repressed by both Mig1 and Mig2 in glucose media, although Mig1 is the main factor in this response [20,26]. We therefore examined biological data on gene expression in *Δmig1 *and *Δmig2 *strains [20,26]. In these studies, 11 genes were presented as being Mig dependent, of which 5 were found in Additional file 1 (*HXK1*, *EMI2*, *HXT13, HXT15 *and *DSF1*) and 3 in Additional file 3 (*REG2*, *DOG2 *and *YLR042C*). We then examined protein binding microarray data [27] and tabulated the combined outputs as Table 4, which lists 15 Mig-dependent genes. When searching the induced set in Additional file 1* in silico *for genes containing Mig1 binding sites, 31 were identified, predominantly containing consensus sequences of the SUC2B type (CCCCGGAT), but also in some cases sites that were more homologous to SUC2A (sharing the consensus motif CCCC(G/A)(G/C)AT [28]). We noted that the presence of a Mig1 site in a gene correlated with high induction (Fig. 3B) and that overall, our analysis provides a highly complete description of how three transcriptions factors might regulate the 146 induced genes of Additional file 1 (Fig. 3C).

**Table 4:** Biologically-verified data on transcription factors that act on genes also found in Additional file 1: transcript dependence on Mig1.

**ORF**	**GENE**	FACTOR CHANGE IN GENE EXPRESSION FROM V5 TO V5.TM6*P^a^	**Mig1 BINDING VERIFIED USING DATA FROM PROTEIN-BINDING MICROARRAY^b ^**[27]** AND *IN VIVO*^c ^**[26]** ANALYSES**
**Glycolysis**			

*YFR053C*	*HXK1*	9	b,c

**Gluconeogenesis**			

*YLR377C*	*FBP1*	5.3	b

**Alternative carbon source utilization**			

*YHL032C*	*GUT1*	6.2	b
*YIL162W*	*SUC2*	6.8	c

**Glyoxylate cycle**			

*YCR005C*	*CIT2*	2.2	b

**Plasma membrane transport**			

*YEL069C*	*HXT13*	3.9	b,c
*YDL245C*	*HXT15*	3.2	c
*YBR298C*	*MAL31*	3.1	b

**Mitochondrial transport**			

*YNR002C*	*ATO2*	3.8	b
*YKL217W*	*JEN1*	10.3	b
*YPL134C*	*ODC1*	6.3	b

**Transcriptional regulation**			

*YDR516C*	*EMI2*	3.6	b,c
*YEL066W*	*HPA3*	2.2	b

**Other**			

*YEL070W*	*DSF1*	9.4	b,c
*YGR243W*	*FMP43*	4.9	b

The table is arranged as for Table 2. Factor changes denoted '^a^' are from Additional file 1. Mig1 binding was verified using data from protein-binding microarray [27] studies denoted '^b' ^and *in vivo *[26] analyses denoted '^c' ^although no factor changes were indicated in those studies.

We noted that *HAP4 *(factor change 1.7, Additional file 3) is induced and *CAT8 *(0.5, adjusted p value < 0.06; Additional file 5), *MIG2 *(0.5, Additional file 1) and *MIG3 *(0.3, Additional file 1) are down-regulated in V5.TM6*P compared to V5. In our dataset, the specific change in array signal for *MIG1 *had an adjusted p-value > 0.05 over the complete experimental range. The result for *CAT8 *was unexpected as it contains an upstream Mig1 binding site similar to *HAP4 *(which is induced) and Cat8 target genes such as *FBP1 *and *PCK1 *are themselves induced. It is possible that the transcript was not probed accurately on the array either due to cross-hybridisation between closely related DNA sequences or on account of its low base expression value (0.24). A future real time Q-PCR experiment would probe the individual transcript.

### Transcripts dependent on external glucose concentration are not found in the respiratory V5.TM6*P strain, even though several glucose- or carbon-source-associated transcripts are glucose dependent in the parental wild-type strain

**Figure 4:**
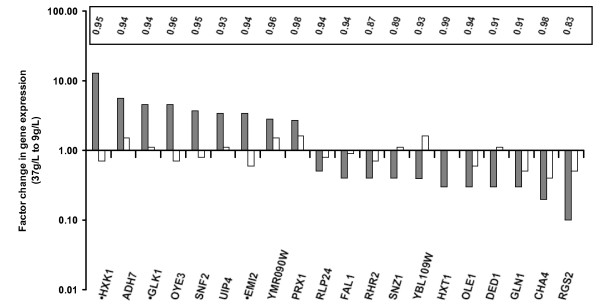
**Transcript dependence on external glucose concentration for 20 genes in the V5 strain**. A clear glucose-dependence (grey bars) was observed for all genes in the V5 strain with only a small variation in array signal (white bars) for the same genes in V5.TM6*P. Genes marked with a dot also have a significant change of expression on going from V5 to V5.TM6*P and as such are listed in Additional file 1. The factor change in gene expression was obtained as the quotient between the expression values at 37 and 9 g/L glucose given by the line fitted to the expression data for each gene. R^2 ^values are for the linear fit for the V5 strain. The R^2 ^values for V5.TM6*P were between 0.16 and 0.78 indicating no significant linear relationship.

The transcriptome of the respiratory V5.TM6*P strain was not found to be responsive to changes in glucose concentration in the culture medium. However, in the wild-type parent strain (V5) the transcriptome was found to vary with glucose availability according to a least squares fit of the logarithmic array values for RNA extracted at the six different glucose concentrations. This dependence on external glucose concentrations is seen in the V5 strain for 20 genes (including those without functional annotations) with an adjusted p-value < 0.05 (Fig. 4). The genes with the strongest dependence on external glucose concentrations were found to be *HXK1*, *RGS2*, *ADH7*, *CHA4 *and *GLK1 *(Fig. 4). The sugar kinases *HXK1 *and *GLK1 *are induced as expected as they have their maximal expression during growth on other carbon sources [16]. The gene product of *RGS2 *inhibits Gpa2 in the PKA pathway, which is one of the pathways induced by glucose [29]. *ADH7 *appears to be induced on carbon sources other than glucose [30]. In a study by DeRisi and colleagues, batch yeast cultures were harvested for cDNA array analysis at gradually decreasing glucose concentration [5]. Our examination of the supplementary data provided by the authors showed that as glucose was consumed between 18.7 and 7.5 g/L, three of the genes in Figure 4 (*GLK1*, *HXK1*, and *FAL1*) were found in the top 22 genes with a changed expression from that study. Overall, the results in Figure 4 show that glucose repression is gradually relieved on going from 37 g/L to 9 g/L glucose. Furthermore relief of repression is already apparent at a surprisingly high glucose concentration, in contrast to the assumption that it is typically triggered over a range of low glucose concentrations [29], the trigger being very low for some genes [31].

In order to examine whether the glucose dependence of genes in V5 affected our data in Additional file 1, we excluded the data points at 13.5 and 5.6 g/L glucose for V5.TM6*P, and 10.5 and 7.4 g/L glucose for V5 (which gave the highest array signals) and compared the cDNA array data in this case with each other exactly as for the full data set. Following this procedure, only a small subset of genes that are highly induced had their factor changes increased substantially, but this did not affect the fact that they remain within the top 30 most induced genes in Additional file 1. They are *HXK1 *(9.0 in Additional file 1, 19.3 following re-calculation; the gene contains binding sites for the Hap complex, Cat8 and Mig1); *STL1 *(21.7, 27.5; Hap complex, Cat8); *DSF1 *(9.4, 15.1; Hap complex, Mig1); *CYB2 *(10.1, 13.4; Hap complex, Mig1) and *FBP1 *(5.3, 7.4; Cat8, Mig1). Fourteen of the seventeen genes that were most affected by this recalculation were found to contain a Mig1 binding site. It is therefore possible that Mig1 is also involved in relieving glucose repression in the 10 to 5 g/L range, and not only at the depletion of glucose at the diauxic shift. Overall, we found that the amplitudes of the factor changes varied by only 9.3% in this new calculation compared with the original one, which indicated that the glucose dependence of the V5 strain does not have a major influence on the way we generated the data presented in Additional file 1.

## Discussion

The main physiological change in both the diauxic shift and the transition from a respiro-fermentative to a respiratory metabolism is that glycolysis is channelled to support respiration: this occurs upon glucose depletion during the diauxic shift and throughout the growth curve in glucose medium in V5.TM6*P. Although previously-published genomic analyses of yeast's response to the diauxic shift transition have all been sampled under different conditions – rich (YPD) medium with 2% glucose [5], minimal defined medium in chemostat cultures at different dilution rates [4], and minimal defined media in batch cultures sampled 4 h after the diauxic shift [3] – common themes emerge with changes on going from V5 to V5.TM6*P. Overall 81% of genes which are induced or repressed on going from V5 to V5.TM6*P in our study were found in at least one of these three datasets for the diauxic shift. Furthermore, we also find that for all proteins induced in a study of the diauxic shift by 2D-gel electrophoresis [6], the corresponding genes are induced on going from V5 to V5.TM6*P except for the alcohol dehydrogenase, Adh2. There is a hence close agreement between induction of the following genes and the levels of the corresponding proteins: *FBP1*, *ICL1*, *PCK1*, *IDP2*, *MLS1*, *DLD1*, *ALD6*, *SDH1 *and *CIT2*. This strong correlation with previously-published data and our own observed correlation of data for Cdc19 and *CDC19 *supports the phenotypic similarity between the V5 to V5.TM6*P transition and the diauxic shift. It is worth noting that there is no clear correlation of our data in Additional file 1 with any possible differences in growth rate between the two strains. In a study by Regenberg and colleagues [32], 180 genes changed in expression by more than a factor of 4 when comparing growth rates. Of these 180, 3% of all genes are found in Additional file 1 providing no significant overlap in data.

It is also possible to see parallels between our data set and a study of nitrogen deprivation and stationary phase growth for wild-type yeast by Gasch and colleagues [33]. This is supported by the fact that ten of the 15 genes that were found to be strongly induced upon nitrogen deprivation and growth in stationary phase in that study are also induced in the V5 to V5.TM6*P transition. Only two genes, *YGR067c *(likely to encode a transcription factor as it has high partial sequence homology with *MIG3 *and *ADR1 *and it fine-tunes the response to nutrient limitation) and *ECM13*, are not induced in the V5.TM6*P strain, but were highly induced in the Gasch study [33] upon nitrogen deprivation and stationary phase growth. In addition, we find that the genes encoding Mep1 and Ato2, which are involved in ammonia transport are also strongly induced in V5.TM6*P in our study. Such an induction is seen in both the diauxic shift studies performed in defined media [4,34], but not in rich medium [5], suggestive of nutrient limitation. In fact, V5.TM6*P also appears carbon limited, not least as a result of the fact that amongst the most strongly induced genes are *FBP1 *and *PCK1*, which encode gluconeogenic enzymes. *STL1*, *MAL31 *and *SUC2 *(encoding proteins involved in usage and/or transport of alternative carbon sources: glycerol, maltose and sucrose respectively) are induced in V5.TM6*P, also consistent with its low glycolytic flux and suggestive of an adaptation of metabolism ('discontinuous modelling) as seen by Brauer and colleagues [4,34] and supported by our previous work [1]. Several of these genes including *FBP1 *and *STL1*, as well as *JEN1*, *ODC1, SFC1 *and *ATO2 *have already been identified as being either induced under carbon limitation or being specific indicators of carbon limitation in yeast cultures [13], consistent with our own data.

The analysis of transcription factor binding sites present in the genes of Additional file 1 revealed that 127 genes had Hap complex binding sites, the majority of which encode mitochondrial proteins. Of genes that only had Hap complex binding sites present, thirteen had only one and of these only five were induced leading us to hypothesise that multiple Hap complex binding sites are correlated with induction of the Additional file 1 genes (Fig. 3A). Interestingly, in a microarray screen of a *hap1Δ *mutant for Hap1-dependent genes [35], 7/24 genes that were induced in the *hap1Δ *mutant are also induced in Additional file 1 (*CYB2*, *MLS1*, *HMX1*, *PUT4*, *STL1*, *HPA2*, *PUT1*), and in each case also have a Hap complex binding site. In addition, 19 genes containing a Cat8 site and 31 genes containing a Mig1 site were predicted, with many of these genes being experimentally-verified in previous *in vivo *studies. Our analysis of the regulation of 88% of the induced genes of our unique respiratory strain (Fig. 3) is further supported by a novel computational method [36] that predicts the Hap complex and Mig1 control the genes of Additional file 1; Cat8 is not included in that database.

A quite unexpected result from comparing the transcriptional profiles of V5 with V5.TM6*P is the data set in Figure 4, showing genes from the parent strain that are clearly glucose dependent and that the glucose dependence observed begins at concentrations (6 – 36 g/L glucose) higher than the range generally regarded as the glucose-repressed region. Strain DBY7286 [5], which is unrelated to V5, previously showed a gradual relief of glucose repression starting at 20 g/L glucose for *GLK1*, *HXK1 *and *FAL1*. These results for V5 and DBY7286 suggest that gradual relief is a general *S. cerevisiae *phenomenon. Future studies will show whether the gradient of the slope is different for different strains. That such glucose dependence requires an intact glucose uptake system is suggested by the fact that it is not seen in the V5.TM6*P strain: the array signal is not changed significantly for any of the genes in the V5.TM6*P strain at the different glucose concentrations sampled. This implies that in V5.TM6*P, glucose sensing is independent of external glucose concentration. The wild-type yeast, on the other hand, shows an extensive modulation of its expression profile at high glucose concentrations despite the fact that its physiological response is largely unaffected. The insensitivity of the V5.TM6*P strain therefore makes it an extremely valuable biotechnological tool as it can be cultured in a wide range of external glucose concentrations, whilst maintaining the same respiratory phenotype [2].

## Conclusion

In this study, we have been able to characterize the transcriptome of a unique respiratory yeast strain. We have been able to identify highly complete collections of known genes in the TCA cycle, glyoxylate cycle and respiratory chain that are consistent with a respiratory metabolism. Our results suggest that there has been genetic remodelling predominantly through the activity of Hap4, Cat8 and Mig1, and that the gene expression profile of V5.TM6*P during growth on glucose resembles wild-type *S. cerevisiae *cells in the diauxic shift.

## Methods

### Yeast strains

The auxotrophic wine yeast strain V5 (*MAT*a *ura3 gal*) and the V5 *hxt1–hxt7*Δ mutant strain (*MAT*a *ura3 gal hxt514*Δ::loxP *hxt367*Δ::lox P *hxt2*Δ::loxP) were kindly provided by the Professor Bruno Blondin, Montpellier, France. The construction of V5.TM6*P was previously described by Henricsson and colleagues [2] where the expression cassette from KOY.TM6*P [1] was transferred into the genome of the V5 *hxt1–hxt7*Δ strain by using the primers PROHXT3 (TCAAATGGCGGTGTAGTTTGAAAAG) and TERHXT7 (TTAAGTGACGGGCGATGAGTAAGAA). Transformants were selected by growth on glucose. The resulting *ura3 *strain is referred to as V5.TM6*. The auxotrophic V5 wild type and V5.TM6* used in this study were made prototrophic by integration of *URA3 *[37] and are referred to as the V5 wild-type strain and V5.TM6*P, respectively.

### Growth conditions

For both pre-cultures and main cultures, 5 × defined minimal medium [38] with 5% glucose as the sole carbon and energy source was used. A two-step pre-cultivation was performed on a rotary shaker. First a 10 mL culture was grown for 72 h, and used to inoculate 100 mL medium, which after 30 h at 30°C was inoculated to give a final OD_610 _of 0.05 in a bioreactor (BRO2, Belach Bioteknik AB, Sweden) in a volume of 2.5 L. Polypropylene glycol P2000 was added as antifoam (100 *μ*L/L). The cultivation conditions were 30°C, 1,000 rpm, pH 5.0 with an airflow of 1.25 L/min. Gas evolution was monitored on-line (type CP460 O_2_/CO_2_, Belach Bioteknik AB). Cultivations were performed at least in duplicate.

### RNA preparation

Yeast cells (2 – 20 mL) were directly transferred into twice their volume of ice-cold water. The cells were collected by centrifugation at 0°C for 5 min at 3000 × g, frozen in liquid nitrogen and stored at -80°C. Total RNA was then prepared using the RNeasy kit from Qiagen, following the manufacturer's instructions.

### cDNA array production

PCR products for the yeast ORFs were generated from yeast DNA using the ORF-specific primer set from Research Genetics and then re-amplified with the Resgen universal forward and reverse primers (Catalogue number 40612). PCR products were prepared by Randy Strich (Fox Chase Cancer Centre, USA) as part of a collaboration with the Wistar Institute, Philadelphia, USA. PCR products were spotted with a BioRobotics Microgrid TASII (Genomic Solutions) by the Wistar Genomics Facility. Each array contained 6,319 individual yeast genes and 1,169 gene repeats. The complete list of arrayed genes on array YA04 is listed under GEO Series accession number GPL4423.

### RNA amplification, hybridization, and scanning

Amplified RNA (aRNA) was prepared from 1 *μ*g of total RNA for each sample tested using the RiboAmp kit (Arcturus). Labelled targets were prepared from 1.6 *μ*g aRNA with Superscript II reverse transcriptase (Invitrogen), in the presence of 3,000–5,000 Ci/mM [α-^33^P] dCTP (Amersham Pharmacia Biotech), 1 mmol/L dATP, 1 mmol/L dTTP, 1 mmol/L dGTP, 67 ng/*μ*g oligo-dT (Promega Biosciences), and 0.65 × random decamer primers (Ambion). Labelled targets were hybridized to individual arrays at 42°C for 18 h in 3 mL Microhyb buffer (Invitrogen). Arrays were washed twice in 2 × standard saline citrate (SSC)/1% SDS solution for 30 min at 50°C, once in 0.5 × SSC/1% SDS and once in 0.1 × SSC/0.5% SDS for 30 min at 55°C. The arrays were exposed to phosphor screens (Amersham Biosciences) for 6 days and scanned in a Storm 820 PhosphorImager (Molecular Dynamics). Quantitation of each spot was assessed by Imagene 5.0 software (BioDiscovery Incorporated) using manual spot alignment, measuring median pixel intensity for each spot and subtracting the local background. The data have been deposited in the NCBI Gene Expression Omnibus and are accessible through GEO Series accession number GSE11799.

### cDNA array data analysis

Observed values less than 0.1 (5% (8752 values) of the 176640 raw data values) were set to 0.1, in accordance with information from Wistar that variations in values below this threshold should be regarded as measurement noise. After converting to a logarithmic scale, average expression values were computed over spots with the same ORF, and arrays were scale normalized by subtracting the median for each of the 24 arrays. Averages were computed over repeated measurements with the same conditions producing 6 values at different glucose concentrations for each of the V5 (38.4, 35, 25.5, 24.4, 10.5, 7.4 g/L glucose) and V5.TM6*P (36.5, 34.8, 26.3, 25.1, 13.5, 5.6 g/L glucose) strains for each gene. T-tests were then performed, comparing the two strains, using Empirical Bayes robustification of variance estimates [39,40].

The T-tests were adjusted for multiple testing using the false discovery rate method (disregarding correlations between genes). Essentially, then, p-values were adjusted so that when selecting all genes with p-values less than a threshold q, a proportion of q false positives would be expected amongst these genes. The estimated effects are shown as factors in Additional file 1, as the linear modelling was performed on a log scale. The p-values in Additional file 1 are the adjusted values, so that selecting genes with p-values < 0.05 gives an expected false discovery rate of 5%. Calculations were performed using the BioConductor package limma [40]. To present the data, a tabular format was chosen over heatmaps in an attempt to make it as accessible as possible to the scientific community.

When producing data for Figure 4, a similar linear model was used, but with a different design matrix: Essentially, for each gene, 2 straight lines were simultaneously fitted, one for each of the strains, to plots of the 6 log-expression values versus glucose concentration. Empirical Bayes robustification and adjustment for multiple testing was included even in this analysis, using the limma tool [40]. Lines with slopes significantly different from zero were found for 20 genes for the V5 strain, but for no genes from the V5.TM6*P strain.

## Authors' contributions

NB initiated the study, prepared the RNA and participated in the data analysis and interpretation, particularly of the transcription factor binding sites. CF carried out the fermentations. PM performed the array analysis. MW performed the statistical analyses of the array data. CC and LS generated the arrays and performed the arraying. LG and CL participated in the experimental design and helped to draft the manuscript. RB participated in the study design, coordinated the data analysis and interpretation, and drafted the manuscript. All authors contributed to the final version of the manuscript.

## Supplementary Material

Additional file 1 Transcriptome analysis of V5.TM6*P compared with the parental V5 strain. Genes were tabulated if their expression was changed on going from the V5 parental strain to V5.TM6*P, as described in 'Materials and Methods'. All genes were then sorted according to their roles in yeast cellular physiology and alphabetically by gene name under each sub-heading. Note that *HXT1-7 *are deleted in the V5.TM6*P with a part of *HXT1 *and *HXT7 *reincorporated as the TM6* chimera. Due to overall high homology within the hexose transporter family we still observed changes on the cDNA array (*HXT1 *(*YHR094C*); factor 0.4; *HXT4 *(*YHR092C*); 0.2; *HXT6 *(*YDR343C*); 2.1; these are not listed in Additional file 2). As YLL053C is annotated as continuous with YLL052C (AQY2) in some strains, these ORFs are counted once here as YLL052C. Genes that were dubious were not included in Additional file 1 and neither were the Ty-transposable elements, *YGR161C-C *(encoding TyA gag protein) and *YNL054W-B *(encoding TyB gag protein). The top 30 genes with the largest numerical factor change (both up- and down-regulated) are underlined. If no functional information was available, phenotypic data from a deletion mutant was entered under 'Function'. In the 'Factor Change' column, change is expressed as a factor, where that factor is x when a gene expressed with intensity '1' in V5 is expressed with intensity 'x' in V5.TM6*P. All T-tests were jointly adjusted for multiple testing using the false discovery rate method (disregarding correlations between genes): p-values were adjusted so that when selecting all genes with p-values less than a threshold q, a proportion of q false positives would be expected amongst these genes. The genes shown have p-values < 0.05, and thus the expected false discovery rate is 5%. Transcription factors are listed if a binding site was predicted as described in the text. Data on 'Copies/cell' are from the yeast GFP fusion localization database and were collected from wild-type yeast grown on glucose. 'Protein Localisation' was extracted from the *Saccharomyces *Genome Database on 14^th ^February 2008 and in each case has been manually curated by the site's curators unless the entry is underlined, indicating that the localization has been extracted from a genome wide study. Click here for file

Additional file 2 This table provides the information from Additional file 1, but in Excel format, with base expression values as well as SAGE [41] and other literature data also included. In the 'Factor Change' column, change is expressed as a factor, where that factor is x when a gene expressed with intensity '1' in V5 is expressed with intensity 'x' in V5.TM6*P. In Additional file 1 we have removed dubious genes and Ty-elements, but all genes that were originally spotted on the array are included in Additional file 2. As for Additional file 1, *YLL052C *and *YLL053C *are treated as one data point. Click here for file

Additional file 3 A further 636 genes changed their expression levels either up by a factor of <2.0 (317 genes) or down by a factor > 0.5 (319 genes) and are presented in Excel format. Click here for file

Additional file 4 Binding sites are listed for the Hap complex, Cat8 and Mig1. The file lists exact position and sequences of the relevant sites. Click here for file

Additional file 5 A computation in which the datasets with the two lowest glucose values have been removed (V5: 10.5, 7.4 g/L glucose and V5.TM6*P: 13.5, 5.6 g/L glucose). Genes are only listed if they are not present in Additional files 1, 2, or 3. Overall, the amplitudes of the factor changes varied by only 9.3% in this new calculation compared with the original one, which indicated that the glucose dependence of the V5 strain does not have a major influence on the way we generated the data presented in Additional file 1. Click here for file
