# Supplementary material for: Transcriptome analysis of a respiratory Saccharomyces cerevisiae strain suggests the expression of its phenotype is glucose insensitive and predominantly controlled by Hap4, Cat8 and Mig1
Source: BMC Genomics. 2008 Jul 31;9:365. doi: 10.1186/1471-2164-9-365 (PMC2536679; doi:10.1186/1471-2164-9-365)
Supplement: Additional file 1 — Transcriptome analysis of V5.TM6*P compared with the parental V5 strain. Genes were tabulated if their expression was changed on going from the V5 parental strain to V5.TM6*P, as described in 'Materials and Methods'. All genes were then sorted according to their roles in yeast cellular physiology and alphabetically by gene name under each sub-heading. Note that HXT1-7 are deleted in the V5.TM6*P with a part of HXT1 and HXT7 reincorporated as the TM6* chimera. Due to overall high homology within the hexose transporter family we still observed changes on the cDNA array (HXT1 (YHR094C); factor 0.4; HXT4 (YHR092C); 0.2; HXT6 (YDR343C); 2.1; these are not listed in Additional file 2). As YLL053C is annotated as continuous with YLL052C (AQY2) in some strains, these ORFs are counted once here as YLL052C. Genes that were dubious were not included in Additional file 1 and neither were the Ty-transposable elements, YGR161C-C (encoding TyA gag protein) and YNL054W-B (encoding TyB gag protein). The top 30 genes with the largest numerical factor change (both up- and down-regulated) are underlined. If no functional information was available, phenotypic data from a deletion mutant was entered under 'Function'. In the 'Factor Change' column, change is expressed as a factor, where that factor is x when a gene expressed with intensity '1' in V5 is expressed with intensity 'x' in V5.TM6*P. All T-tests were jointly adjusted for multiple testing using the false discovery rate method (disregarding correlations between genes): p-values were adjusted so that when selecting all genes with p-values less than a threshold q, a proportion of q false positives would be expected amongst these genes. The genes shown have p-values < 0.05, and thus the expected false discovery rate is 5%. Transcription factors are listed if a binding site was predicted as described in the text. Data on 'Copies/cell' are from the yeast GFP fusion localization database and were collected from wild-type yeast grown on [file 1471-2164-9-365-S1.pdf]

ORF	GENE	FUNCTION	FACTOR CHANGE	TRANSCRIPTION FACTOR BINDING SITES PREDICTED FOR Hap4, Cat8 and Mig1	COPIES OF PROTEIN PER CEL	PROTEN LOCALISATION
**Glycolysis, fermentation and related reactions**						
YAL038W	CDC19	Pyruvate kinase, functions as a homotetramer in glycolysis to convert phosphoenolpyruvate to pyruvate, the input for aerobic (TCA cycle) or anaerobic (glucose fermentation) respiration	0.4		291000	cytosol
YCL040W	GLK1	Glucokinase, catalyzes the phosphorylation of glucose at C6 in the first irreversible step of glucose metabolism; one of three glucose phosphorylating enzymes; expression regulated by non-fermentable carbon sources	3.8	Mig1	21100	cytosol
YFR053C	HXK1	Hexokinase isoenzyme 1, a cytosolic protein that catalyzes phosphorylation of glucose during glucose metabolism; expression is highest during growth on non-glucose carbon sources; glucose-induced repression involves the hexokinase Hxk2	9.0	Cat8 Mig1	40800	cytosol
YLR044C	PDC1	Major of three pyruvate decarboxylase isozymes, key enzyme in alcoholic fermentation, decarboxylates pyruvate to acetaldehyde; subject to glucose-, ethanol-, and autoregulation; involved in amino acid catabolism	0.2		8970	nucleus; cytosol
YLR134W	PDC5	Minor isoform of pyruvate decarboxylase, key enzyme in alcoholic fermentation, decarboxylates pyruvate to acetaldehyde, regulation is glucose- and ethanol-dependent, repressed by thiamine, involved in amino acid catabolism	0.2		471000	nucleus; cytosol
YOR347C	PYK2	Pyruvate kinase, one of two isoforms that catalyze the final step in glycolysis; activity appears to be modulated by phosphorylation; PYK2 transcription is repressed by glucose, and Pyk2 may be active under low glycolytic flux	2.0		2130	mitochondrion; cytosol
**Gluconeogenesis**						
YLR377C	FBP1	Fructose-1,6-bisphosphatase, key regulatory enzyme in the gluconeogenesis pathway, required for glucose metabolism	5.3	Cat8 Mig1	-	cytosol
YKR097W	PCK1	Phosphoenolpyruvate carboxykinase, key enzyme in gluconeogenesis, catalyzes early reaction in carbohydrate biosynthesis, glucose represses transcription and accelerates mRNA degradation, regulated by Mcm1 and Cat8, located in the cytosol	6.9	Cat8 Mig1	-	cytosol
**Alternative carbon source utilization**						
YPL061W	ALD6	Cytosolic aldehyde dehydrogenase that is activated by Mg^{2+} and utilizes $NADP^+$ as the preferred coenzyme; required for the conversion of acetaldehyde to acetate; constitutively expressed; locates to the mitochondrial outer surface upon oxidative stress	2.3	Hap4 Cat8	135000	mitochondrion; cytosol
YAL060W	BDH1	NAD-dependent (R,R)-butanediol dehydrogenase, catalyzes oxidation of (R,R)-2,3-butanediol to (3R)-acetoin, oxidation of meso-butanediol to (3S)-acetoin, and reduction of acetoin; enhances use of 2,3-butanediol as an aerobic carbon source	3.0	Mig1	8730	cytoplasm
YAL061W	BDH2	Putative medium-chain alcohol dehydrogenase with similarity to BDH1; transcription induced by constitutively active PDR1 and PDR3; BDH2 is an essential gene	2.9		3090	nucleus; cytoplasm
YML054C	CYB2	Cytochrome b2 (L-lactate cytochrome-c oxidoreductase), component of the mitochondrial intermembrane space, required for lactate utilization; expression is repressed by glucose and anaerobic conditions	10.1	Hap4 Mig1	-	mitochondrial intermembrane space
YEL071W	DLD3	D-lactate dehydrogenase, part of the retrograde regulon which consists of genes whose expression is stimulated by damage to mitochondria and reduced in cells grown with glutamate as the sole nitrogen source, located in the cytoplasm	0.5		13000	cytoplasm soluble fraction
YHL032C	GUT1	Glycerol kinase, converts glycerol to glycerol-3-phosphate; glucose repression of expression is mediated by Adr1 and Ino2-Ino4; derepression of expression on non-fermentable carbon sources is mediated by Opi1 and Rsf1	6.2	Hap4 Mig1	-	cytoplasm
YIL155C	GUT2	Mitochondrial glycerol-3-phosphate dehydrogenase; expression is repressed by both glucose and cAMP and derepressed by non-fermentable carbon sources in a Snf1, Rsf1, Hap2/3/4/5 complex dependent manner	3.3	Hap4	1670	Integral to mitochondrial outer membrane; mitochondrion
YIL162W	SUC2	Invertase, sucrose hydrolyzing enzyme; a secreted, glycosylated form is regulated by glucose repression, and an intracellular, nonglycosylated enzyme is produced constitutively	6.8	Mig1	-	extracellular region; mitochondrion; cytoplasm
YGL157W	-	Oxidoreductase, catalyzes NADPH-dependent reduction of the bicyclic diketone bicyclo[2.2.2]octane-2,6-dione (BCO2,6D) to the chiral ketoalcohol (1R,4S,6S)-6-hydroxybicyclo[2.2.2]octane-2-one (BCO2one6ol)	0.3		2950	Cytoplasm; nucleus
**TCA cycle**						
YLR304C	ACO1	Aconitase, required for the tricarboxylic acid (TCA) cycle and also independently required for mitochondrial genome maintenance; phosphorylated;component of the mitochondrial nucleoid; mutation leads to glutamate auxotrophy	3.2	Hap4	96700	cytosol; mitochondrial matrix; mitochondrial nucleoid
YJL200C	ACO2	Putative mitochondrial aconitase isozyme; similarity to Aco1, an aconitase required for the TCA cycle; expression induced during growth on glucose, by amino acid starvation via Gcn4, and repressed on ethanol	0.5	Hap4	4670	mitochondrion
YNR001C	CIT1	Citrate synthase, catalyzes the condensation of acetyl coenzyme A and oxaloacetate to form citrate; the rate-limiting enzyme of the TCA cycle; nuclear encoded mitochondrial protein	3.0	Hap4	-	mitochondrion
YPL262W	FUM1	Fumarase, converts fumaric acid to L-malic acid in the TCA cycle; cytosolic and mitochondrial localization determined by the N-terminal mitochondrial targeting sequence and protein conformation; phosphorylated in mitochondria	3.8	Hap4	6920	mitochondrial matrix; cytosol
YNL037C	IDH1	Subunit of mitochondrial NAD^+-dependent isocitrate dehydrogenase, which catalyzes the oxidation of isocitrate to alpha-ketoglutarate in the TCA cycle	3.1	Hap4	10500	mitochondrial nucleoid; mitochondrial matrix
YIL125W	KGD1	Component of the mitochondrial alpha-ketoglutarate dehydrogenase complex, which catalyzes the oxidative decarboxylation of alpha-ketoglutarate to form succinyl-CoA in the TCA cycle	2.2	Hap4	14300	mitochondrial nucleoid; mitochondrial matrix
YDR148C	KGD2	Dihydrolipoyl transsuccinylase, a component of the mitochondrial alpha-ketoglutarate dehydrogenase complex, which catalyzes a step in the tricarboxylic acid (TCA) cycle, the oxidative decarboxylation of alpha-ketoglutarate to succinyl-CoA	3.5	Hap4	7970	mitochondrial nucleoid; mitochondrial matrix
YFL018C	LPD1	Dihydrolipoamide dehydrogenase, the lipoamide dehydrogenase component (E3) of the pyruvate dehydrogenase and 2-oxoglutarate dehydrogenase multi-enzyme complexes	2.0	Hap4	24600	mitochondrial nucleoid;
YGR244C	LSC2	Beta subunit of succinyl-CoA ligase, which is a mitochondrial enzyme of the TCA cycle that catalyzes the nucleotide-dependent conversion of succinyl-CoA to succinate	2.9	Hap4	-	mitochondrion
YKL085W	MDH1	Mitochondrial malate dehydrogenase, catalyzes interconversion of malate and oxaloacetate; involved in the tricarboxylic acid (TCA) cycle; phosphorylated	3.6	Hap4	28100	mitochondrial matrix
YKL148C	SDH1	Flavoprotein subunit of succinate dehydrogenase (Sdh1, Sdh2, Sdh3, Sdh4), which couples the oxidation of succinate to the transfer of electrons to ubiquinone	4.6	Hap4	-	mitochondrial respiratory chain complex II
YLL041C	SDH2	Iron-sulfur protein subunit of succinate dehydrogenase (Sdh1, Sdh2, Sdh3, Sdh4), which couples the oxidation of succinate to the transfer of electrons to ubiquinone	4.1	Hap4	9540	mitochondrial respiratory chain complex II
YKL141W	SDH3	Cytochrome b subunit of succinate dehydrogenase (Sdh1, Sdh2, Sdh3, Sdh4), which couples the oxidation of succinate to the transfer of electrons to ubiquinone	3.7	Hap4	-	mitochondrial respiratory chain complex II
YDR178W	SDH4	Membrane anchor subunit of succinate dehydrogenase (Sdh1, Sdh2, Sdh3, Sdh4), which couples the oxidation of succinate to the transfer of electrons to ubiquinone	5.3	Hap4 Mig1	7920	mitochondrial respiratory chain complex II
**Glyoxylate cycle and related reactions**						
YCR005C	CIT2	Citrate synthase, catalyzes the condensation of acetyl coenzyme A and oxaloacetate to form citrate, peroxisomal isozyme involved in glyoxylate cycle; expression is controlled by Rtg1 and Rtg2 transcription factors	2.2	Hap4 Cat8 Mig1	2310	peroxisome mitochondrion
YIR029W	DAL2	Allantoicase, converts allantoate to urea and ureidoglycolate in the second step of allantoin degradation; expression sensitive to nitrogen catabolite repression and induced by allophanate, an intermediate in allantoin degradation	3.0	Hap4	-	-
YLR174W	IDP2	Cytosolic NADP-specific isocitrate dehydrogenase, catalyzes oxidation of isocitrate to alpha-ketoglutarate; levels are elevated during growth on non-fermentable carbon sources and reduced during growth on glucose	4.0	Hap4 Cat8	-	cytosol
YOL126C	MDH2	Cytoplasmic malate dehydrogenase, one of the three isozymes that catalyze interconversion of malate and oxaloacetate; involved in gluconeogenesis during growth on ethanol or acetate as carbon source; interacts with Pck1 and Fbp1	3.0	Hap4 Cat8	5260	cytosol
YNL117W	MLS1	Malate synthase, enzyme of the glyoxylate cycle, involved in utilization of non-fermentable carbon sources; expression is subject to carbon catabolite repression; localizes in peroxisomes during growth in oleic acid medium	3.5	Hap4 Cat8	-	peroxisomal matrix

**Respiratory chain**						
YBL099W	*ATP1*	Alpha subunit of the F_1 sector of mitochondrial F_1F_0 ATP synthase, which is a large, evolutionarily conserved enzyme complex required for ATP synthesis; phosphorylated	2.4	Hap4	41500	mitochondrial nucleoid; mitochondrion
YBR039W	*ATP3*	Gamma subunit of the F_1 sector of mitochondrial F_1F_0 ATP synthase, which is a large, evolutionarily conserved enzyme complex required for ATP synthesis	2.5	Hap4	28100	mitochondrial proton-transporting ATP synthase, central stalk; mitochondrion
YPL078C	*ATP4*	Subunit b of the stator stalk of mitochondrial F_1F_0 ATP synthase, which is a large, evolutionarily conserved enzyme complex required for ATP synthesis	2.1	Hap4	12900	mitochondrial proton-transporting ATP synthase, stator stalk; mitochondrion
YKL016C	*ATP7*	Subunit d of the stator stalk of mitochondrial F_1F_0 ATP synthase, which is a large, evolutionarily conserved enzyme complex required for ATP synthesis	2.3	Hap4	6820	mitochondrial proton-transporting ATP synthase, stator stalk; mitochondrion
YPL271W	*ATP15*	Epsilon subunit of the F_1 sector of mitochondrial F_1F_0 ATP synthase, which is a large, evolutionarily conserved enzyme complex required for ATP synthesis; phosphorylated	2.2	Hap4	4280	mitochondrial proton-transporting ATP synthase, central stalk; mitochondrion
YDL004W	*ATP16*	Delta subunit of the central stalk of mitochondrial F_1F_0 ATP synthase, which is a large, evolutionarily conserved enzyme complex required for ATP synthesis;phosphorylated	2.0	Hap4	-	mitochondrial proton-transporting ATP synthase, central stalk; mitochondrion
YOL077W-A	*ATP19*	Subunit k of the mitochondrial F_1F_0 ATP synthase, which is a large enzyme complex required for ATP synthesis; associated only with the dimeric form of ATP synthase	2.5	Hap4	1320	mitochondrial proton-transporting ATP synthase complex, coupling factor F_0; mitochondrion
YPR020W	*ATP20*	Subunit g of the mitochondrial F_1F_0 ATP synthase, reversibly phosphorylated on two residues; unphosphorylated form is required for dimerization of the ATP synthase complex	2.5	Hap4	Low signal	mitochondrial proton-transporting ATP synthase complex, coupling factor F_0; mitochondrion
YGR174C	*CBP4*	Mitochondrial protein required for assembly of ubiquinol cytochrome-c reductase complex (cytochrome bc1 complex); interacts with Cbp3 and function is partially redundant with that of Cbp3	2.8	Hap4	704	Integral to mitochondrial membrane; mitochondrion
YBL045C	*COR1*	Core subunit of the ubiquinol-cytochrome c reductase complex (bc1 complex), which is a component of the mitochondrial inner membrane electron transport chain	2.0	Hap4	19300	mitochondrial respiratory chain complex III; mitochondrion
YGL187C	*COX4*	Subunit IV of cytochrome c oxidase, which is the terminal member of the mitochondrial inner membrane electron transport chain; N-terminal 25 residues of precursor are cleaved during mitochondrial import; phosphorylated	2.0	Hap4 Cat8	9410	mitochondrial respiratory chain complex IV; mitochondrion
YNL052W	*COX5A*	Subunit Va of cytochrome c oxidase, which is the terminal member of the mitochondrial inner membrane electron transport chain; predominantly expressed during aerobic growth while its isoform Vb (Cox5Bp) is expressed during anaerobic growth	2.0	Hap4	3670	mitochondrial respiratory chain complex IV; mitochondrion
YHR051W	*COX6*	Subunit VI of cytochrome c oxidase, which is the terminal member of the mitochondrial inner membrane electron transport chain; expression is regulated by oxygen levels	2.1	Hap4	12500	mitochondrial respiratory chain complex IV; mitochondrion
YLR038C	*COX12*	Subunit VIb of cytochrome c oxidase, which is the terminal member of the mitochondrial inner membrane electron transport chain; required for assembly of fully active cytochrome c oxidase but not required for activity after assembly; phopshorylated	2.4	Hap4	1390	mitochondrial respiratory chain complex IV; mitochondrion
YLL009C	*COX17*	Copper metallochaperone that transfers copper to Sco1 and Cox11 for eventual delivery to cytochrome c oxidase	2.8	Hap4	-	cytosol; mitochondrial intermembrane space
YDR231C	*COX20*	Mitochondrial inner membrane protein, required for proteolytic processing of Cox2 and its assembly into cytochrome c oxidase	2.1	Hap4	Low signal	mitochondrial inner membrane
YOR065W	*CYT1*	Cytochrome c1, component of the mitochondrial respiratory chain; expression is regulated by the heme-activated, glucose-repressed Hap2/3/4/5 CCAAT-binding complex	2.5	Hap4 Mig1	39900	mitochondrial inner membrane; mitochondrial respiratory chain complex III; mitochondrion
YKL087C	*CYT2*	Cytochrome c1 heme lyase, involved in maturation of cytochrome c1, which is a subunit of the mitochondrial ubiquinol-cytochrome-c reductase; links heme covalently to apocytochrome c1	2.2	Hap4	-	mitochondrial intermembrane space
YIL098C	*FMC1*	Mitochondrial matrix protein, required for assembly or stability at high temperature of the F_1 sector of mitochondrial F_1F_0 ATP synthase; null mutant temperature sensitive growth on glycerol is suppressed by multicopy expression of Odc1	2.5	Hap4	589	mitochondrion
YGL040C	*HEM2*	Delta-aminolevulinate dehydratase, a homo-octameric enzyme, catalyzes the conversion of delta-aminolevulinic acid to porphobilinogen, the second step in the heme biosynthetic pathway; localizes to both the cytoplasm and nucleus	2.2	Hap4	11600	nucleus; cytoplasm
YLR205C	*HMX1*	ER localized, heme-binding peroxidase involved in the degradation of heme; does not exhibit heme oxygenase activity despite similarity to heme oxygenases; expression regulated by *AFT1*	2.4	Hap4	-	endoplasmic reticulum membrane
YOR020C	*HSP10*	Mitochondrial matrix co-chaperonin that inhibits the ATPase activity of Hsp60, a mitochondrial chaperonin; involved in protein folding and sorting in the mitochondria; 10 kD heat shock protein with similarity to *E. coli* GroES	2.1	Hap4	-	mitochondrial matrix
YDL181W	*INH1*	Protein that inhibits ATP hydrolysis by the F_1F_0-ATP synthase, inhibitory function is enhanced by stabilizing proteins Stf1 and Stf2; has similarity to Stf1 and both Inh1 and Stf1 exhibit the potential to form coiled-coil structures	2.2	Hap4	981	mitochondrial proton-transporting ATP synthase complex; mitochondrion
YBR185C	*MBA1*	Protein involved in assembly of mitochondrial respiratory complexes; may act as a receptor for proteins destined for export from the mitochondrial matrix to the inner membrane	2.3	Hap4	2720	mitochondrial inner membrane
YML120C	*NDI1*	NADH:ubiquinone oxidoreductase, transfers electrons from NADH to ubiquinone in the respiratory chain but does not pump protons, in contrast to the higher eukaryotic multisubunit respiratory complex I; phosphorylated; homolog of human AMID	4.1	Hap4	5240	mitochondrial matrix
YMR267W	*PPA2*	Mitochondrial inorganic pyrophosphatase, required for mitochondrial function and possibly involved in energy generation from inorganic pyrophosphate	2.3	Hap4	195	mitochondrion
YDR529C	*QCR7*	Subunit 7 of the ubiquinol cytochrome-c reductase complex, which is a component of the mitochondrial inner membrane electron transport chain; oriented facing the mitochondrial matrix; N-terminus appears to play a role in complex assembly	2.2	Hap4	10100	mitochondrial respiratory chain complex III; mitochondrion
YHR001W-A	*QCR10*	Subunit of the ubiqunol-cytochrome c oxidoreductase complex and comprises part of the mitochondrial respiratory chain	2.5	Hap4	5590	mitochondrial respiratory chain complex III; mitochondrion
YEL024W	*RIP1*	Ubiquinol-cytochrome-c reductase, a Rieske iron-sulfur protein of the mitochondrial cytochrome bc1 complex; transfers electrons from ubiquinol to cytochrome c1 during respiration	2.3	Hap4	-	mitochondrial respiratory chain complex III; mitochondrion
YGR008C	*STF2*	Protein involved in regulation of the mitochondrial F_1F_0-ATP synthase; Stf1 and Stf2 act as stabilizing factors that enhance inhibitory action of the Inh1 protein	3.1	Hap4	5330	mitochondrial proton-transporting ATP synthase complex
YPR151C	*SUE1*	Mitochondrial protein required for degradation of unstable forms of cytochrome c	3.7	Hap4	-	mitochondrial envelope
YDR322C-A	*TIM11*	Subunit e of mitochondrial F_1F_0-ATPase, which is a large, evolutionarily conserved enzyme complex required for ATP synthesis; essential for the dimeric and oligomeric state of ATP synthase	3.0	Hap4	4590	mitochondrial proton-transporting ATP synthase complex, coupling factor F_0;mitochondrion
**Oxidative stress response**						
YLR109W	*AHP1*	Thiol-specific peroxiredoxin, reduces hydroperoxides to protect against oxidative damage; function in vivo requires covalent conjugation to Urm1	0.4	Hap4	16200	cytoplasm
YML060W	*OGG1*	Mitochondrial glycosylase/lyase that specifically excises 7,8-dihydro-8-oxoguanine residues located opposite cytosine or thymine residues in DNA, repairs oxidative damage to mitochondrial DNA	2.2	Hap4	3690	mitochondrion
YGR209C	*TRX2*	Cytoplasmic thioredoxin isoenzyme of the thioredoxin system which protects cells against both oxidative and reductive stress, forms *LMA1* complex with Pbi2, acts as a cofactor for Tsa1, required for ER-Golgi transport and vacuole inheritance	0.5		17200	vacuole; cytosol
YGR234W	*YHB1*	Nitric oxide oxidoreductase, flavohemoglobin involved in nitric oxide detoxification; plays a role in the oxidative and nitrosative stress responses	2.4	Hap4	13000	cytosol; mitochondrial matrix
**Plasma membrane transport**						
YLL052C	*AQY2*	Water channel that mediates the transport of water across cell membranes and may be involved in freeze tolerance; disrupted by a stop codon in many *S. cerevisiae* strains	3.0	Hap4	Low signal	Endoplasmic reticulum membrane
YML116W	*ATR1*	Multidrug efflux pump of the major facilitator superfamily, required for resistance to aminotriazole and 4-nitroquinoline-N-oxide	0.4		1720	plasma membrane
YDR046C	*BAP3*	Amino acid permease involved in the uptake of cysteine, leucine, isoleucine and valine	0.4	Hap4	Low signal	plasma membrane
YLR411W	*CTR3*	High-affinity copper transporter of the plasma membrane, acts as a trimer; gene is disrupted by a Ty2 transposon insertion in many laboratory strains of *S. cerevisiae*	3.0		-	integral to plasma membrane

YOL158C	*ENB1*	Endosomal ferric enterobactin transporter, expressed under conditions of iron deprivation; member of the major facilitator superfamily; expression is regulated by Rcs1 and affected by chloroquine treatment	0.4	Hap4	-	cytoplasmic membrane-bound vesicle; endosome; integral to membrane
YDR508C	*GNP1*	High-affinity glutamine permease, also transports Leu, Ser, Thr, Cys, Met and Asn; expression is fully dependent on Grr1 and modulated by the Ssy1-Ptr3-Ssy5 (SPS) sensor of extracellular amino acids	0.4		10600	plasma membrane
YEL069C	*HXT13*	Hexose transporter, induced in the presence of non-fermentable carbon sources, induced by low levels of glucose, repressed by high levels of glucose	3.9	Hap4 Mig1	-	plasma membrane
YDL245C	*HXT15*	Protein of unknown function with similarity to hexose transporter family members, expression is induced by low levels of glucose and repressed by high levels of glucose	3.2	Mig1	-	plasma membrane
YJR158W	*HXT16*	Protein of unknown function with similarity to hexose transporter family members, expression is repressed by high levels of glucose	3.5	Mig1	-	plasma membrane
YBR298C	*MAL31*	Maltose permease, high-affinity maltose transporter (alpha-glucoside transporter); encoded in the *MAL3* complex locus; member of the 12 transmembrane domain superfamily of sugar transporters; functional in genomic reference strain S288C	3.1	Mig1	-	membrane fraction
YGR121C	*MEP1*	Ammonium permease; belongs to a ubiquitous family of cytoplasmic membrane proteins that transport only ammonium (NH4+); expression is under the nitrogen catabolite repression regulation	2.3		-	plasma membrane
YOR348C	*PUT4*	Proline permease, required for high-affinity transport of proline; also transports the toxic proline analog azetidine-2-carboxylate (AzC); *PUT4* transcription is repressed in ammonia-grown cells	5.8	Hap4 Cat8	-	plasma membrane
YDR536W	*STL1*	Glycerol proton symporter of the plasma membrane, subject to glucose-induced inactivation, strongly but transiently induced when cells are subjected to osmotic shock	21.7	Hap4 Cat8	-	plasma membrane; membrane
YNL160W	*YGP1*	Cell wall-related secretory glycoprotein; induced by nutrient deprivation-associated growth arrest and upon entry into stationary phase; may be involved in adaptation prior to stationary phase entry; has similarity to Sps100	2.6	Hap4	-	cell wall
YGL255W	*ZRT1*	High-affinity zinc transporter of the plasma membrane, responsible for the majority of zinc uptake; transcription is induced under low-zinc conditions by the Zap1 transcription factor	0.5		-	Integral to plasma membrane
**Mitochondrial transport**						
YMR056C	*AAC1*	Mitochondrial inner membrane ADP/ATP translocator, exchanges cytosolic ADP for mitochondrially synthesized ATP; Aac1 is a minor isoform while Pet9 is the major ADP/ATP translocator	2.5	Hap4	768	mitochondrial inner membrane
YNR002C	*ATO2*	Putative transmembrane protein, involved in the export of ammonia, a starvation signal that promotes cell death in the center of aging colonies; member of the TC 9.B.33 YaaH family; homolog of Ady2 and *Y. lipolytica* Gpr1	3.8	Hap4 Mig1	-	plasma membrane
YML042W	*CAT2*	Carnitine acetyl-CoA transferase present in both mitochondria and peroxisomes, transfers activated acetyl groups to carnitine to form acetylcarnitine which can be shuttled across membranes	2.6	Hap4 Cat8	-	mitochondrion; peroxisome; peroxisomal matrix
YOR100C	*CRC1*	Mitochondrial inner membrane carnitine transporter, required for carnitine-dependent transport of acetyl-CoA from peroxisomes to mitochondria during fatty acid beta-oxidation	2.2	Hap4 Cat8	-	mitochondrial inner membrane
YKL217W	*JEN1*	Lactate transporter, required for uptake of lactate and pyruvate; phosphorylated; expression is derepressed by transcriptional activator Cat8 during respiratory growth and repressed in the presence of glucose, fructose, and mannose	10.3	Hap4 Cat8 Mig1	-	plasma membrane; mitochondrion
YPL134C	*ODC1*	Mitochondrial inner membrane transporter, exports 2-oxoadipate and 2-oxoglutarate from the mitochondrial matrix to the cytosol for lysine and glutamate biosynthesis and lysine catabolism; suppresses, in multicopy, an fmc1 null mutation	6.3	Hap4 Cat8 Mig1	2840	mitochondrion; mitochondrial inner membrane
YNL055C	*POR1*	Mitochondrial porin (voltage-dependent anion channel), outer membrane protein required for the maintenance of mitochondrial osmotic stability and mitochondrial membrane permeability	3.1	Hap4	-	mitochondrion; integral to mitochondrial outer membrane
YJR095W	*SFC1*	Mitochondrial succinate-fumarate transporter, transports succinate into and fumarate out of the mitochondrion; required for ethanol and acetate utilization	18.3	Hap4 Cat8 Mig1	-	mitochondrial inner membrane
**Transcriptional regulation**						
YKL139W	*CTK1*	Catalytic (alpha) subunit of C-terminal domain kinase I (CTDK-I), which phosphorylates the C-terminal repeated domain of the RNA polymerase II large subunit (Rpo21) to affect both transcription and pre-mRNA 3' end processing	0.4	Hap4	1970	nucleus
YDR516C	*EMI2*	Non-essential protein of unknown function required for transcriptional induction of the early meiotic-specific transcription factor IME1; required for sporulation; expression is regulated by glucose-repression transcription factors Mig1/2	3.6	Mig1	10600	cytoplasm
YOL071W	*EMI5*	Non-essential protein of unknown function required for transcriptional induction of the early meiotic-specific transcription factor IME1, also required for sporulation	2.1	Hap4	1080	mitochondrion
YPR193C	*HPA2*	Tetrameric histone acetyltransferase with similarity to Gcn5, Hat1, Elp3, and Hpa3; acetylates histones H3 and H4 in vitro and exhibits autoacetylation activity	4.4	Hap4	-	cytoplasm
YEL066W	*HPA3*	D-Amino acid N-acetyltransferase, catalyzes N-acetylation of D-amino acids through ordered bi-bi mechanism in which acetyl-CoA is first substrate bound and CoA is last product liberated; similar to Hpa2, acetylates histones weakly *in vitro*	2.2	Hap4 Mig1	1160	nucleus; cytoplasm
YGL209W	*MIG2*	Protein containing zinc fingers, involved in repression, along with Mig1, of *SUC2* (invertase) expression by high levels of glucose; binds to Mig1-binding sites in *SUC2* promoter	0.5	Hap4	504	nucleus
YER028C	*MIG3*	Probable transcriptional repressor involved in response to toxic agents such as hydroxyurea that inhibit ribonucleotide reductase; phosphorylation by Snf1 or the Mec1 pathway inactivates Mig3, allowing induction of damage response genes	0.3		-	nucleus
YNL333W	*SNZ2*	Member of a stationary phase-induced gene family; transcription of *SNZ2* is induced prior to diauxic shift, and also in the absence of thiamin in a Thi2-dependent manner; forms a coregulated gene pair with *SNO2*; interacts with Thi11	3.0	Hap4	125	-
YFL059W	*SNZ3*	Member of a stationary phase-induced gene family; transcription of *SNZ2* is induced prior to diauxic shift, and also in the absence of thiamin in a Thi2-dependent manner; forms a coregulated gene pair with *SNO3*	2.8	Hap4	-	-
**Sulfur assimilation**						
YOL064C	*MET22*	Bisphosphate-3'-nucleotidase, involved in salt tolerance and methionine biogenesis; dephosphorylates 3'-phosphoadenosine-5'-phosphate and 3'-phosphoadenosine-5'-phosphosulfate, intermediates of the sulfate assimilation pathway	0.5		7330	cytoplasm
YLL061W	*MMP1*	High-affinity S-methylmethionine permease, required for utilization of S-methylmethionine as a sulfur source; has similarity to S-adenosylmethionine permease Sam3	2.9	Hap4 Mig1	Low signal	plasma membrane
YLL058W	-	Putative protein of unknown function with similarity to Str2, which is a cystathionine gamma-synthase important in sulfur metabolism; *YLL058W* is not an essential gene	0.4	Hap4	-	-
**Fatty acid metabolism**						
YLR056W	*ERG3*	C-5 sterol desaturase, catalyzes the introduction of a C-5(6) double bond into episterol, a precursor in ergosterol biosynthesis; mutants are viable, but cannot grow on non-fermentable carbon sources	2.1	Mig1	36200	endoplasmic reticulum
YLL031C	*GPI13*	ER membrane localized phosphoryltransferase that adds phosphoethanolamine onto the third mannose residue of the glycosylphosphatidylinositol (GPI) anchor precursor; similar to human PIG-O protein	0.5		300	endoplasmic reticulum
YJL134W	*LCB3*	Long-chain base-1-phosphate phosphatise with specificity for dihydrosphingosine-1-phosphate, regulates ceramide and long-chain base phosphates levels, involved in incorporation of exogenous long chain bases in sphingolipids	0.5		-	endoplasmic reticulum
YKL150W	*MCR1*	Mitochondrial NADH-cytochrome b5 reductase, involved in ergosterol biosynthesis	3.1	Hap4	1920	integral to mitochondrial outer membrane; mitochondrial intermembrane space
YAR042W	*SWH1*	Protein similar to mammalian oxysterol-binding protein; contains ankyrin repeats; localizes to the Golgi and the nucleus-vacuole junction	0.3		846	early endosome; endoplasmic reticulum; Golgi trans cisterna ; nuclear envelope

**Biosynthesis**						
YFL030W	*AGX1*	Alanine : glyoxylate aminotransferase (AGT), catalyzes the synthesis of glycine from glyoxylate, which is one of three pathways for glycine biosynthesis in yeast; has similarity to mammalian and plant alanine : glyoxylate aminotransferases	3.6		339	mitochondrion
YER026C	*CHO1*	Phosphatidylserine synthase, functions in phospholipids biosynthesis; catalyzes the reaction CDP-diaclyglycerol + L-serine = CMP + L-1-phosphatidylserine, transcriptionally repressed by myo-inositol and choline	2.4		-	endoplasmic reticulum
YJL099W	*CHS6*	Member of the ChAPs family of proteins (Chs5-Arf1-binding proteins: Bch1, Bch2, Bud7, Chs6), that forms the exomer complex with Chs5 to mediate export of specific cargo proteins, including Chs3, from the Golgi to the plasma membrane	2.0		1180	exomer complex; colocalizes with trans-Golgi network transport vesicle
YML078W	*CPR3*	Mitochondrial peptidyl-prolyl cis-trans isomerase (cyclophilin), catalyzes the cis-trans isomerization of peptide bonds N-terminal to proline residues; involved in protein refolding after import into mitochondria	2.0	Hap4	1960	mitochondrion
YDR019C	*GCV1*	T subunit of the mitochondrial glycine decarboxylase complex, required for the catabolism of glycine to 5,10-methylene-THF; expression is regulated by levels of levels of 5,10-methylene-THF in the cytoplasm	0.4	Hap4	4090	mitochondrion
YEL011W	*GLC3*	Glycogen branching enzyme, involved in glycogen accumulation; green fluorescent protein (GFP)-fusion protein localizes to the cytoplasm in a punctate pattern	2.5	Mig1	1230	cytoplasm
YER055C	*HIS1*	ATP phosphoribosyltransferase, a hexameric enzyme, catalyzes the first step in histidine biosynthesis; mutations cause histidine auxotrophy and sensitivity to Cu, Co, and Ni salts; transcription is regulated by general amino acid control	0.4		-	intracellular
YOR226C	*ISU2*	Conserved protein of the mitochondrial matrix, required for synthesis of mitochondrial and cytosolic iron-sulfur proteins, performs a scaffolding function in mitochondria during Fe/S cluster assembly; *isu1 isu2* double mutant is inviable	0.4	Hap4	3420	mitochondrial matrix
YLR142W	*PUT1*	Proline oxidase, nuclear-encoded mitochondrial protein involved in utilization of proline as sole nitrogen source; *PUT1* transcription is induced by Put3 in the presence of proline and the absence of a preferred nitrogen source	2.0	Hap4	184	mitochondrion
YOL055C	*THI20*	Multifunctional protein with both hydroxymethylpyrimidine kinase and thiaminase activities; involved in thiamine biosynthesis and also in thiamine degradation; member of a gene family with THI21 and THI22; functionally redundant with Thi21	0.5		195	cytosol
YBR006W	*UGA2*	Succinate semialdehyde dehydrogenase involved in the utilization of gamma-aminobutyrate (GABA) as a nitrogen source; part of the 4-aminobutyrate and glutamate degradation pathways; localized to the cytoplasm	2.2	Mig1	Low signal	cytoplasm
**Ribosomal proteins in the mitochondria or cytosol**						
YGL121C	*GPG1*	Proposed gamma subunit of the heterotrimeric G protein that interacts with the receptor Grp1; involved in regulation of pseudohyphal growth; requires Gpb1 or Gpb2 to interact with Gpa2	2.6	Hap4	-	-
YDR296W	*MHR1*	Protein involved in homologous recombination in mitochondria and in transcription regulation in nucleus; binds to activation domains of acidic activators; required for recombination-dependent mtDNA partitioning	2.0	Hap4	2610	nucleus; mitochondrion
YGL068W	*MNP1*	Protein associated with the mitochondrial nucleoid; putative mitochondrial ribosomal protein with similarity to E. coli L7/L12 ribosomal protein; required for normal respiratory growth	2.1		6940	mitochondrial ribosome
YDR347W	*MRP1*	Mitochondrial ribosomal protein of the small subunit; *MRP1* exhibits genetic interactions with *PET122*, encoding a *COX3*-specific translational activator, and with *PET123*, encoding a small subunit mitochondrial ribosomal protein	2.3	Hap4	2840	mitochondrial small ribosomal subunit
YDR116C	*MRPL1*	Mitochondrial ribosomal protein of the large subunit	2.6	Hap4	7010	mitochondrial large ribosomal subunit
YKL138C	*MRPL31*	Mitochondrial ribosomal protein of the large subunit	2.2	Hap4	2120	mitochondrial large ribosomal subunit
YGR165W	*MRPS35*	Mitochondrial ribosomal protein of the small subunit	2.1	Hap4	3460	mitochondrial small ribosomal subunit
YJR094W-A	*RPL43B*	Protein component of the large (60S) ribosomal subunit, identical to Rpl43Ap and has similarity to rat L37a ribosomal protein	0.4	Hap4	45500	cytosolic large ribosomal subunit
YBR189W	*RPS9B*	Protein component of the small (40S) ribosomal subunit; nearly identical to Rps9Ap and has similarity to *E. coli* S4 and rat S9 ribosomal proteins	0.5		63300	cytosolic small ribosomal subunit
YKL156W	*RPS27A*	Protein component of the small (40S) ribosomal subunit; nearly identical to Rps27Bp and has similarity to rat S27 ribosomal protein	2.5	Hap4	43300	cytosolic small ribosomal subunit
YHR038W	*RRF1*	Mitochondrial ribosome recycling factor, essential for mitochondrial protein synthesis and for the maintenance of the respiratory function of mitochondria	2.2	Hap4	1440	mitochondrion
YDR041W	*RSM10*	Mitochondrial ribosomal protein of the small subunit, has similarity to *E. coli* S10 ribosomal protein; essential for viability, unlike most other mitoribosomal proteins	2.1	Hap4	8480	mitochondrial small ribosomal subunit
YDR175C	*RSM24*	Mitochondrial ribosomal protein of the small subunit	2.4	Hap4	3150	mitochondrial small ribosomal subunit
YHR196W	*UTP9*	Nucleolar protein, component of the small subunit (SSU) processome containing the U3 snoRNA that is involved in processing of pre-18S rRNA	0.5	Hap4	20000	nucleus; rDNA heterochromatin; small subunit processome
YFR049W	*YMR31*	Mitochondrial ribosomal protein of the small subunit, has similarity to human mitochondrial ribosomal protein MRP-S36	2.5	Hap4 Mig1	2050	mitochondrial small ribosomal subunit
**Other**						
YAL046C	*AIM1*	Putative protein of unknown function; null mutant displays increased frequency of mitochondrial genome loss (petite formation)	2.0	Mig1	1710	-
YBR262C	*AIM5*	Protein of unknown function; non-tagged protein is detected in purified mitochondria in high-throughput studies; null mutant displays decreased frequency of mitochondrial genome loss and reduced growth rate in minimal glycerol media	2.3	Hap4	2550	mitochondrion
YFR011C	*AIM13*	Putative protein of unknown function; non-tagged protein is detected in highly purified mitochondria; null mutant displays increased frequency of mitochondrial genome loss (petite formation) and reduced growth rate in minimal glycerol media	2.1	Hap4	3550	mitochondrion; cytoplasm
YJR080C	*AIM24*	Protein of unknown function; non-tagged protein is detected in purified mitochondria in high-throughput studies; null mutant displays increased frequency of mitochondrial genome loss and reduced growth rate in minimal glycerol media	2.1	Hap4	2290	mitochondrion
YLR168C	*AIM30*	Putative protein of unknown function that may be involved in intramitochondrial sorting; similar to Ups1 and to human PRELI; GFP-tagged protein localizes to mitochondria; required for wild-type respiratory growth	2.6	Hap4	2910	mitochondrion
YML030W	*AIM31*	Putative protein of unknown function; GFP-fusion protein localizes to mitochondria; null mutant is viable and displays decreased frequency of mitochondrial genome loss (petite formation) and severe growth rate in minimal glycerol media	2.3	Hap4	1210	mitochondrion
YML087C	*AIM33*	Putative protein of unknown function; null mutant displays increased frequency of mitochondrial genome loss (petite formation) and severe growth defect in minimal glycerol media	4.2	Hap4	-	-
YNL100W	*AIM37*	Putative protein of unknown function; non-tagged protein is detected in purified mitochondria; null mutant displays decreased frequency of mitochondrial genome loss (petite formation) and severe growth defect in minimal glycerol media	2.4	Hap4	5460	mitochondrion
YNR018W	*AIM38*	Putative protein of unknown function; non-tagged protein is detected in purified mitochondria; null mutant displays decreased frequency of mitochondrial genome loss (petite formation) and severe growth defect in minimal glycerol media	2.5	Hap4	12000	mitochondrion
YKR027W	*BCH2*	Member of the ChAPs family of proteins (Chs5-Arf1-binding proteins: Bch1, Bch2, Bud7, Chs6), that forms the exomer complex with Chs5 to mediate export of specific cargo proteins, including Chs3, from the Golgi to the plasma membrane	2.5		2540	exomer complex; colocalizes with trans-Golgi network
YJL014W	*CCT3*	Subunit of the cytosolic chaperonin Cct ring complex, related to Tcp1, required for the assembly of actin and tubulins *in vivo*	0.5	Hap4	-	cytoskeleton; cytoplasm
YBR038W	*CHS2*	Chitin synthase II, requires activation from zymogenic form in order to catalyze the transfer of N-acetylglucosamine (GlcNAc) to chitin; required for the synthesis of chitin in the primary septum during cytokinesis	2.1		-	cellular bud neck
YEL070W	*DSF1*	Deletion suppressor of mpt5 mutation	9.4	Hap4 Mig1	-	-
YGR243W	*FMP43*	Putative protein of unknown function; the authentic, non-tagged protein is detected in highly purified mitochondria in high-throughput studies	4.9	Hap4 Mig1	6750	mitochondrion
YDR110W	*FOB1*	Nucleolar protein required for DNA replication fork blocking and recombinational hotspot activities; binds to the replication fork barrier site in the rDNA region; related to retroviral integrases	0.4		1510	nucleolus
YOL152W	*FRE7*	Putative ferric reductase with similarity to Fre2; expression induced by low copper levels	2.7	Hap4	-	plasma membrane
YJR118C	*ILM1*	Protein of unknown function; may be involved in mitochondrial DNA maintenance; required for slowed DNA synthesis-induced filamentous growth	0.4		1160	endoplasmic reticulum
YFR038W	*IRC5*	Putative ATPase containing the DEAD/H helicase-related sequence motif; null mutant displays increased levels of spontaneous Rad52 foci	0.5		-	-

YKR019C	*IRS4*	Protein involved in regulation of phosphatidylinositol 4,5-bisphosphate concentrations; Irs4 and Tax4 bind and activate the phosphatase Inp51; mutation confers an increase in rDNA silencing	3.7		-	mitochondrion
YHR039C	*MSC7*	Protein of unknown function, green fluorescent protein (GFP)-fusion protein localizes to the endoplasmic reticulum; msc7 mutants are defective in directing meiotic recombination events to homologous chromatids	0.4		2500	endoplasmic reticulum
YPL052W	*OAZ1*	Regulator of ornithine decarboxylase (Spe1), antizyme that binds to Spe1 to regulate ubiquitin-independent degradation; ribosomal frameshifting during synthesis of Oaz1 and its ubiquitin-mediated degradation are both polyamine-regulated	0.5	Hap4	-	-
YBR230C	*OM14*	Integral mitochondrial outer membrane protein; abundance is decreased in cells grown in glucose relative to other carbon sources; appears to contain 3 alpha-helical transmembrane segments; ORF encodes a 97-basepair intron	3.3	Hap4 Cat8	4750	integral to mitochondrial outer membrane
YIL136W	*OM45*	Protein of unknown function, major constituent of the mitochondrial outer membrane; located on the outer (cytosolic) face of the outer membrane	7.3	Hap4 Cat8	6490	integral to mitochondrial outer membrane; mitochondrial outer membrane
YDR329C	*PEX3*	Peroxisomal membrane protein (PMP) required required for the proper localization and stability of PMPs; interacts with Pex19	2.8	Hap4	1400	peroxisomal membrane; endoplasmic reticulum
YKL163W	*PIR3*	O-glycosylated covalently-bound cell wall protein required for cell wall stability; expression is cell cycle regulated, peaking in M/G1 and also subject to regulation by the cell integrity pathway	2.4	Hap4	-	cell wall
YAL023C	*PMT2*	Protein O-mannosyltransferase, transfers mannose residues from dolichyl phosphate-D-mannose to protein serine/threonine residues; acts in a complex with Pmt1, can instead interact with Pmt5 in some conditions; target for new antifungals	0.5		6510	endoplasmic reticulum
YDL039C	*PRM7*	Pheromone-regulated protein, predicted to have one transmembrane segment; promoter contains Gcn4 binding elements	2.3		-	integral to membrane
YGL120C	*PRP43*	RNA helicase in the DEAH-box family, functions in both RNA polymerase I and polymerase II transcript metabolism, involved in release of the lariat-intron from the spliceosome	0.5		16900	spliceosome
YLR204W	*QRI5*	Mitochondrial inner membrane protein, required for accumulation of spliced *COX1* mRNA; may have an additional role in translation of *COX1* mRNA	2.1	Hap4	-	integral to mitochondrial inner membrane
YDL104C	*QRI7*	Putative metalloprotease, similar to O-sialoglycoprotein metallopeptidase from P. haemolytica; the authentic, non-tagged protein is detected in highly purified mitochondria in high-throughput studies	2.1	Hap4	1400	mitochondrion
YGR070W	*ROM1*	GDP/GTP exchange protein (GEP) for Rho1; mutations are synthetically lethal with mutations in rom2, which also encodes a GEP	0.4	Hap4	-	intracellular
YDL048C	*STP4*	Protein containing a Kruppel-type zinc-finger domain; has similarity to Stp1, Stp2, and Stp3	2.0		623	nucleus; mitochondrion; cytoplasm
YJL035C	*TAD2*	Subunit of tRNA-specific adenosine-34 deaminase, forms a heterodimer with Tad3 that converts adenosine to inosine at the wobble position of several tRNAs	0.5		830	nucleus; cytoplasm
YLR327C	*TMA10*	Protein of unknown function that associates with ribosomes	4.6	Hap4 Mig1	504	ribosome
YER049W	*TPA1*	Protein of unknown function; interacts with Sup45 (eRF1), Sup35 (eRF3) and Pab1; has a role in translation termination efficiency, mRNA poly(A) tail length and mRNA stability	0.5	Hap4	8910	nucleus
YOR187W	*TUF1*	Mitochondrial translation elongation factor Tu; comprises both GTPase and guanine nucleotide exchange factor activities, while these activities are found in separate proteins in *S. pombe* and humans	2.3	Hap4	58500	mitochondrial matrix
YFL010C	*WWM1*	WW domain containing protein of unknown function; binds to Mca1, a caspase-related protease that regulates H_2O_2-induced apoptosis; overexpression causes G_i phase growth arrest and clonal death that is suppressed by overexpression of *MCA1*	0.4		6020	nucleus
YKL067W	*YNK1*	Nucleoside diphosphate kinase, catalyzes the transfer of gamma phosphates from nucleoside triphosphates, usually ATP, to nucleoside diphosphates by a mechanism that involves formation of an autophosphorylated enzyme intermediate	3.1		7130	cytosol; mitochondrial intermembrane space
YBR046C	*ZTA1*	Zeta-crystallin homolog, found in the cytoplasm and nucleus; has similarity to E. coli quinone oxidoreductase and to human zeta-crystallin, which has quinone oxidoreductase activity	2.6		-	nucleus; cytoplasm
YAR068W	-	Fungal-specific protein of unknown function; induced in respiratory-deficient cells	0.4		-	-
YEL020C	-	Hypothetical protein with low sequence identity to Pdc1	2.0		-	cytoplasm
YER067W	-	Putative protein of unknown function; green fluorescent protein (GFP)-fusion protein localizes to the cytoplasm and nucleus; YER067W is not an essential gene	4.1	Hap4	8450	cytoplasm; nucleus
YGR110W	-	Putative protein of unknown function; transcription is increased in response to genotoxic stress; plays a role in restricting Ty1 transposition	2.3	Hap4	-	-
YHR151C	-	Putative protein of unknown function	0.5		-	-
YMR034C	-	Putative transporter, member of the SLC10 carrier family; identified in a transposon mutagenesis screen as a gene involved in azole resistance; YMR034C is not an essential gene	2.0		-	-
YOL098C	-	Putative metalloprotease	0.5		8120	cytoplasm
YOL114C	-	Putative protein of unknown function with similarity to human ICT1 and prokaryotic factors that may function in translation termination; YOL114C is not an essential gene	2.1	Hap4	-	-
YOR019W	-	Protein of unknown function that may interact with ribosomes, based on co-purification experiments	2.1	Cat8	-	-
YOR214C	-	Putative protein of unknown function; YOR214C is not an essential gene	0.5		-	cell wall
YOR289W	-	Putative protein of unknown function; transcription induced by the unfolded protein response; green fluorescent protein (GFP)-fusion protein localizes to both the cytoplasm and the nucleus	2.0	Mig1	-	cytoplasm; nucleus
YPR148C	-	Protein of unknown function that may interact with ribosomes, based on co-purification experiments; green fluorescent protein (GFP)-fusion protein localizes to the cytoplasm in a punctate pattern	2.1	Hap4	Low signal	cytoplasm
YPR157W	-	Putative protein of unknown function	2.4		-	-
